# Classifying information-sharing methods

**DOI:** 10.1186/s12874-021-01292-z

**Published:** 2021-05-22

**Authors:** Georgios F. Nikolaidis, Beth Woods, Stephen Palmer, Marta O. Soares

**Affiliations:** 1 The University of York, Centre for Health Economics, Alcuin A Block, Heslington, York, YO10 5DD UK; 2 IQVIA, 210 Pentonville Road, London, N1 9JY UK

**Keywords:** Meta-analysis, Network meta-analysis, Borrowing-strength, Indirect evidence, Information-sharing

## Abstract

**Background:**

Sparse relative effectiveness evidence is a frequent problem in Health Technology Assessment (HTA). Where evidence directly pertaining to the decision problem is sparse, it may be feasible to expand the evidence-base to include studies that relate to the decision problem only indirectly: for instance, when there is no evidence on a comparator, evidence on other treatments of the same molecular class could be used; similarly, a decision on children may borrow-strength from evidence on adults. Usually, in HTA, such indirect evidence is either included by ignoring any differences (‘lumping’) or not included at all (‘splitting’). However, a range of more sophisticated methods exists, primarily in the biostatistics literature. The objective of this study is to identify and classify the breadth of the available information-sharing methods.

**Methods:**

Forwards and backwards citation-mining techniques were used on a set of seminal papers on the topic of information-sharing. Papers were included if they specified (network) meta-analytic methods for combining information from distinct populations, interventions, outcomes or study-designs.

**Results:**

Overall, 89 papers were included. A plethora of evidence synthesis methods have been used for information-sharing. Most papers (*n*=79) described methods that shared information on relative treatment effects. Amongst these, there was a strong emphasis on methods for information-sharing across multiple outcomes (*n*=42) and treatments (*n*=25), with fewer papers focusing on study-designs (*n*=23) or populations (*n*=8). We categorise and discuss the methods under four ’core’ relationships of information-sharing: functional, exchangeability-based, prior-based and multivariate relationships, and explain the assumptions made within each of these core approaches.

**Conclusions:**

This study highlights the range of information-sharing methods available. These methods often impose more moderate assumptions than lumping or splitting. Hence, the degree of information-sharing that they impose could potentially be considered more appropriate. Our identification of four ‘core’ methods of information-sharing allows for an improved understanding of the assumptions underpinning the different methods. Further research is required to understand how the methods differ in terms of the strength of sharing they impose and the implications of this for health care decisions.

**Supplementary Information:**

The online version contains supplementary material available at (10.1186/s12874-021-01292-z).

## Background

Health Technology Assessment (HTA) is the systematic evaluation of the properties, effects and impact of health technologies with a view to inform decision-making in health care [1]. Regardless of whether or not a system functions under explicit budget constraints, resources spent could have always been used for alternative purposes. Therefore, policy-makers are always faced with difficult decisions about whether interventions should be funded. This requires an assessment of whether the benefits of an intervention are sufficient to justify the health opportunity costs of funding it [2]. It follows that a set of tools ought to be used so that policy-makers can rationally and transparently decide about the adoption of a given health technology [3].

Decision analysis provides a quantitative framework that brings together all relevant evidence on the impact of an intervention on health outcomes and costs, whilst making explicit judgements about how different types and sources of evidence are linked together (model structure) and which elements are relevant to decision-making (reflecting social values). The outputs of a Decision Analytic Model (DAM) include incremental costs and benefits and can be useful for decision-makers [4].

Each input within a DAM is a parameter and constitutes a potential research question that can be informed by evidence which is typically identified using literature reviews. To assist study selection when identifying evidence for reviews, research questions are defined using the PICOS framework, where P stands for Population, I for Intervention, C for Comparator, O for Outcome, and S for Study-design [5]. Typically, reviewers exclude studies deviating from the inclusion criteria on any PICOS dimension; that is, they usually only include studies providing *direct evidence*. Hence, direct evidence on relative effectiveness comprises of one or more randomised studies, evaluating the intervention(s) under assessment, recruiting patients from the population of interest, and measuring effects on all relevant outcomes.

Where multiple studies exist to inform the same parameter, these can be synthesised to generate a single estimate that represents the evidence-base. To synthesise the evidence base and provide DAMs with relative effectiveness inputs, standard Meta-Analysis (MA) and Network Meta-Analysis (NMA) methods [6, 7] are commonly used. Although synthesis is more common for Relative Treatment Effects (RTEs), evidence synthesis methods can also be applied for other DAM inputs such as costs and Quality of Life (QoL).

However, in HTA, direct evidence may be sparse, heterogeneous, or limited in other ways and synthesis may become problematic. Where evidence is sparse, it may not be possible to obtain the required Relative Treatment Effect (RTE) estimates, and even when they can be obtained, they may be highly uncertain and may not be robust due to assumptions imposed in the analysis [8, 9]. Evidence sparsity may also prevent appropriate exploration of heterogeneity because small studies are at higher risk of enrolling unrepresentative populations [10] and provide less evidence to enable robust subgroup analyses.

**Fig. 1:**
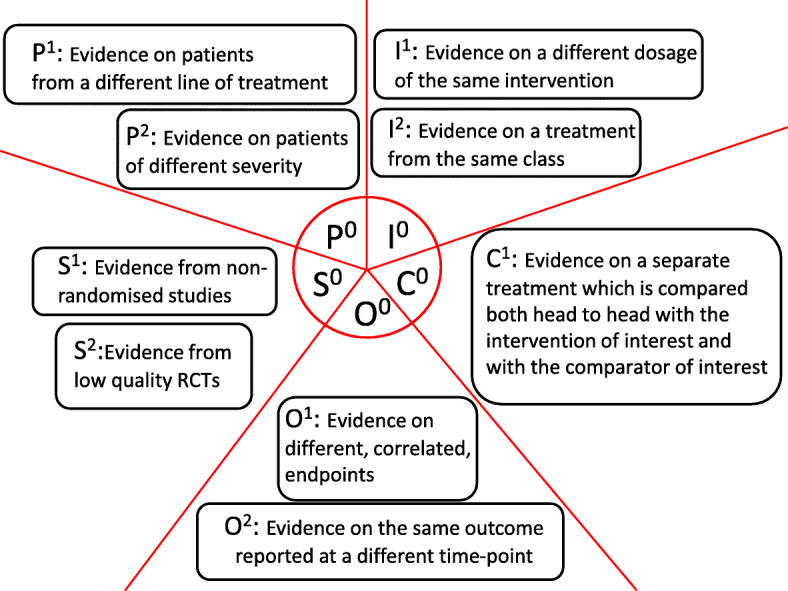
An illustration of the extended evidence base. The small pie in the middle, characterised by *P*^0^,*I*^0^,*C*^0^,*O*^0^,*S*^0^, represents only the directly relevant information which usually comprise only a small part of the evidence that is relevant to a decision. The evidence outside the small pie represent examples of indirectly relevant information for each of the PICOS dimensions

A policy relevant alternative to limited or sparse data may be to extend the evidence base beyond the direct evidence. A topical example concerns paediatric indications for which the evidence-base is typically sparse due to the regulatory restrictions on trials. To support decision-making for this population, the Food and Drugs Administration (FDA) [11] and the European Medicines Agency (EMA) now propose that *“The evidence needed to address the research questions that are important for marketing authorisation of a given product in the target population might be modified based on what is known for other populations”* [12]. Whilst in the aforementioned example the evidence is extended to consider another population, in principle, indirect evidence may relate to any other dimension of PICOS (Fig. 1) —it may include studies assessing a different, but related, treatment or pertaining to a different study-design than what is specified in the research question. Note that, in this context, NMA also considers indirect evidence, pertaining to other treatment comparisons i.e. indirect evidence on the ‘Intervention’ PICOS dimension, to inform the treatment effect(s) of primary interest [13].

Within a decision-making context, the use of indirect evidence, as long as it is judged relevant, contributes to accountability by allowing for all relevant evidence to be considered. Combining all relevant sources of evidence may yield more precise estimates than the direct evidence alone and allow better characterisation of heterogeneity and uncertainty. However, when indirect evidence are not sufficiently relevant or of high-quality, using indirect evidence may also introduce bias and inflate heterogeneity estimates.

The use of indirect evidence to support decision-making is not exclusive to the aforementioned regulatory context and has permeated HTA processes. Examples can be found in Technology Appraisals (TAs) conducted by the National Institute for Health and Care Excellence (NICE) to inform routine use of technologies in the National Health Service (NHS) in England and Wales. For instance, TA445 [14] considered adult studies to complement a sparse paediatric evidence base. Also, relative effectiveness has been generalised across subgroups of different Hepatitis C genotypes [15]. These two examples use indirect evidence by considering both sources perfectly generalisable (‘lumping’), as an alternative to being considered completely independent (‘splitting’). There are, however, examples of appraisals which use indirect evidence in more sophisticated ways. For instance, TA383 [16] used indirect evidence across interventions by assuming a ‘class-effect‘ between treatments that function through the same molecular pathway. TA139 [17] and TA168 [18] simultaneously modelled two outcomes leveraging their correlation structure and TA244 [19] modelled a network of interventions with multiple treatment components assuming that the relative effect of an intervention is the sum of the relative effects of its comprising components.

Inevitably, a judgement on whether the indirect evidence is relevant is always required. However, what is often not made explicit is that, where both direct and indirect evidence are considered, there should be appropriate consideration for the extent of information-sharing permitted by different synthesis methods (i.e. the extent to which the indirect evidence is allowed to affect the estimates obtained by using only the direct evidence).

The objective of this review is to identify information-sharing evidence synthesis methods that have been used in the literature and improve understanding of these methods by making explicit the fundamental assumptions underpinning them. We do so by identifying the ‘core’ relationships used to share information. This review increases awareness around the breadth of available information-sharing methods and aids transparency in information-sharing methods choice. To our knowledge, this topic has not been explored in the past with a clear policy focus.

## Methods

We conducted a literature search, that was systematic and transparent in its methods and conduct, but was not comprehensive due to the challenges described ahead [20]. We, therefore, will refer to our methodology as a literature review and not as a systematic review.

To inform the design and conduct of the literature review, we initially conducted a scoping review (details provided in Additional file 1). It’s aims were to clarify working definitions, determine inclusion and exclusion criteria, understand whether keyword-based methods [20] would generate a sensitive and specific search strategy, obtain a comprehensive list of representative seminal papers on information-sharing, and conceive how the breadth of information-sharing methods could be categorised in a useful manner. We found that consistent terminology was not used when referring to methods that combined direct and indirect evidence. Therefore, for the main literature review, we used citation-mining methods [21] which are efficient [22] and have been used for similar reviews [23]. The methods of the main review were protocolled in advance.

Citation-mining involves two steps. The first encompassed the compilation of a list of seminal/influential papers. All relevant papers identified in the scoping review were considered and seminal papers were selected to reflect breadth [22] by including different fields of research: MA, NMA, multi-parameter evidence synthesis, synthesis of multiple outcomes and the incorporation of evidence on historical controls in trial-design. Two external evidence synthesis experts were consulted to validate the list and provide additional references. The second, and main, step of the citation-mining review was then conducted in the Web of Science (WoS) on 20/Feb/2019 by identifying all the citations of the seven seminal papers [8, 24–29], and then all articles that cited the seminal papers — i.e. a forwards and backwards citation-mining.

Inclusion and exclusion criteria were pre-specified (see Additional file 1). Articles were included if they formally specified MA or NMA models (in mathematical notation or computer code) that combined information pertaining to multiple populations, interventions, outcomes or study-designs, or if they utilised evidence from an external source (such as a previous meta-analysis). Given the aim of identifying a range of methods for the sharing of information, papers that used only standard NMA methods originally described by Lu et al. [7] (i.e. by pooling evidence sets assuming perfect exchangeability) were excluded.

Data extraction was pre-specified and included year of publication, the synthesis challenge addressed, the specific method (relationship) imposed between the ‘direct’ and ‘indirect’ evidence to facilitate information-sharing, the PICOS dimension(s) of indirectness, the ‘cores’ used, the parameter over which information-sharing was imposed, and whether the paper fell into the field of MA or NMA. When papers tackled multiple challenges simultaneously (e.g. [30–32]), the challenges they dealt with were isolated and extracted separately. Further information on the search strategy and inclusion and exclusion criteria is provided in Additional file 1.

The search was conducted in Zotero version 5.0.69 and a link to the included papers Zotero database, where the papers have been grouped according to various tags, is provided in the end of the manuscript. The PRISMA checklist for systematic reviews is provided in Additional file 2. The results of the search were reported descriptively, by grouping the methods by the policy problem and the PICOS dimension of indirectness. Methods were then categorised according to the ‘core’ relationship they used to enable information-sharing and the statistical methods falling within each core were described.

## Results

### Characteristics of the included studies

**Fig. 2:**
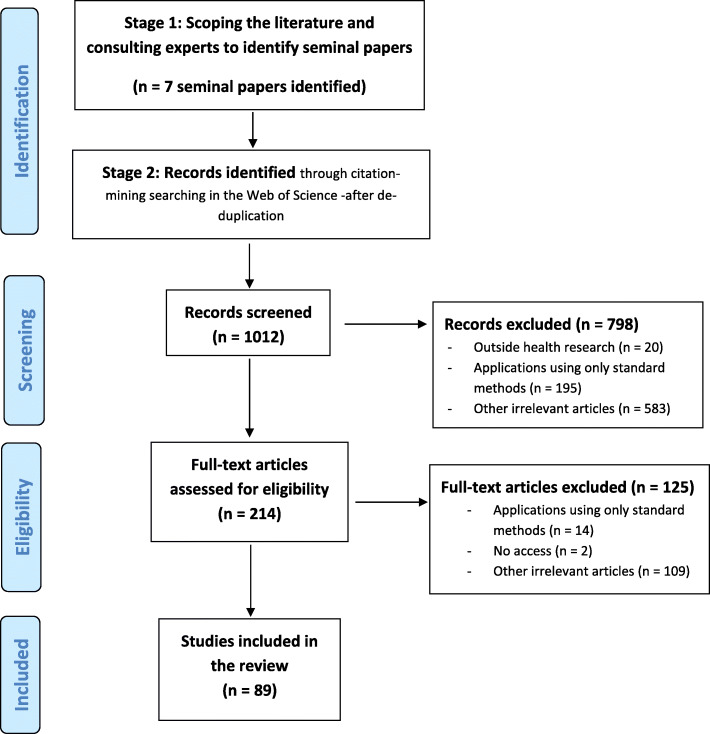
PRISMA diagram for search results

The review identified 89 papers (Fig. 2) which are available in our online database (link provided in the end of the manuscript). The majority (*n*=79) described methods that shared information on relative treatment effects. Other studies used methods to share information on comparison-specific meta-regression slopes (*n*=4), comparison-specific between-studies heterogeneities (*n*=6), or study-specific baselines (*n*=2). Overall, there was a balance amongst papers that developed methods within MA (*n*=45) and NMA (*n*=44). There was a strong emphasis on methods for information-sharing across multiple outcomes (42 papers) and treatments (25 papers), with fewer papers focusing on study-designs (23 papers) or populations (8 papers) (Table 1). Note that some papers described methods sharing information on several types of parameters and across more than one PICOS dimension (e.g. [30–32]). A full list of the included papers along with a description of how information was shared within each paper can be found in Additional file 3.

**Table 1:** A categorisation of papers that shared information according to the ‘core’ relationship that they used and the PICOS dimension that direct and indirect evidence differ in

PICOS dimension of indirectness	‘Core‘ relationship			
	Functional	Exchangeability based	Prior-based	Multivariate
Intervention	Lumping: [30, 32–43]	RE: [34, 38–42]	SIP: No refs	B: [36]
	C: [35, 39, 40, 44]	RW: [35]	MixP: No refs	W: No refs
	L: [30, 35–38, 42, 45–48]	MLM: [32, 33, 35, 44, 46, 49, 50]	PP: No refs	BW: No refs
	N-L: [35, 51–53]			S: No refs
Population	Lumping: [14, 33]	RE: [34, 54]	SIP: [55]	B: No refs
	C: No refs	RW: No refs	MixP: [56]	W: No refs
	L: [33]	MLM: [55]	PP: [55]	BW: No refs
	N-L: No refs			S: No refs
Outcomes	Lumping: [32]	RE: No refs	SIP: No refs	B: [30, 31, 57–60]
	C: [57]	RW: [45, 61]	MixP: No refs	W: [62]
	L: [31]	MLM: [30]	PP: No refs	BW: [26, 63–78]
	N-L: [61, 79]			S: [80–84]
Designs	Lumping: No refs	RE: No refs	SIP: [85–90]	B: No refs
	C: No refs	RW: No refs	MixP: No refs	W: No refs
	L: [42, 50, 90–99]	MLM: [85, 86, 88, 100]	PP: [101]	BW: No refs
	N-L: No refs			S: No refs

The ‘PICOS dimension of indirectness’ denotes the PICOS part (i.e. Population, Intervention etc.) on which the direct evidence differ from the indirect in terms of the research question they address.

C: Constraint, L: Linear relationship (e.g. meta-regression), N-L: Non-linear, RE: Random-Effect, RW: Random-Walk, MLM: Multi-level model, SIP: Standard Informative Prior, MixP: Mixture prior, PP: Power-prior, B: Only between-studies correlation modelled, W: Only within-study correlation modelled, B&W: Both within-study and between-studies correlations modelled separately, S: Within-study and between-studies correlations modelled simultaneously as one parameter

We also identified the most common synthesis challenges addressed by the included papers. Amongst papers sharing information across populations, 3 [14, 55, 56] developed models to accommodate simultaneous synthesis of adult and paediatric evidence, 1 [33] described models that allowed information-sharing between patients subgroups, and 2 [34, 54] provided model extensions for baseline risk adjustment. Amongst papers sharing information across interventions, 7 [35, 44–46, 51–53] simultaneously synthesised multiple dosages of the same treatment, 7 [32, 33, 36, 44, 46, 49, 50] shared information across interventions that fall under the same ‘class’, 5 [30, 36, 37, 47, 48] dealt with complex interventions (i.e. treatments that comprise multiple components), and 4 [38–41] described models on comparison-specific non-relative effect parameters such as between-studies variances or meta-regression slopes. Amongst papers sharing information across study-designs, 9 [8, 85–89, 100–102] combined randomised and non-randomised studies and 13 [42, 50, 85, 90–99] dealt with studies’ internal or external biases. Amongst papers that shared information across outcomes, 2 [31, 57] considered structurally related outcomes (for instance, when one outcome has to occur before the other), 6 [30, 45, 58, 59, 61, 79] combined evidence from studies that reported at multiple/different follow-up periods, and 34 papers [8, 25, 26, 30, 31, 57, 60, 62–78, 80–84, 102–106] considered correlated outcomes. Finally, 7 papers described how evidence from previous meta-analyses (meta-epidemiological evidence) [24, 90, 107–109] or expert elicitation [68, 110] can be incorporated in analyses.

### ‘Core’ relationships for information-sharing

**Fig. 3:**
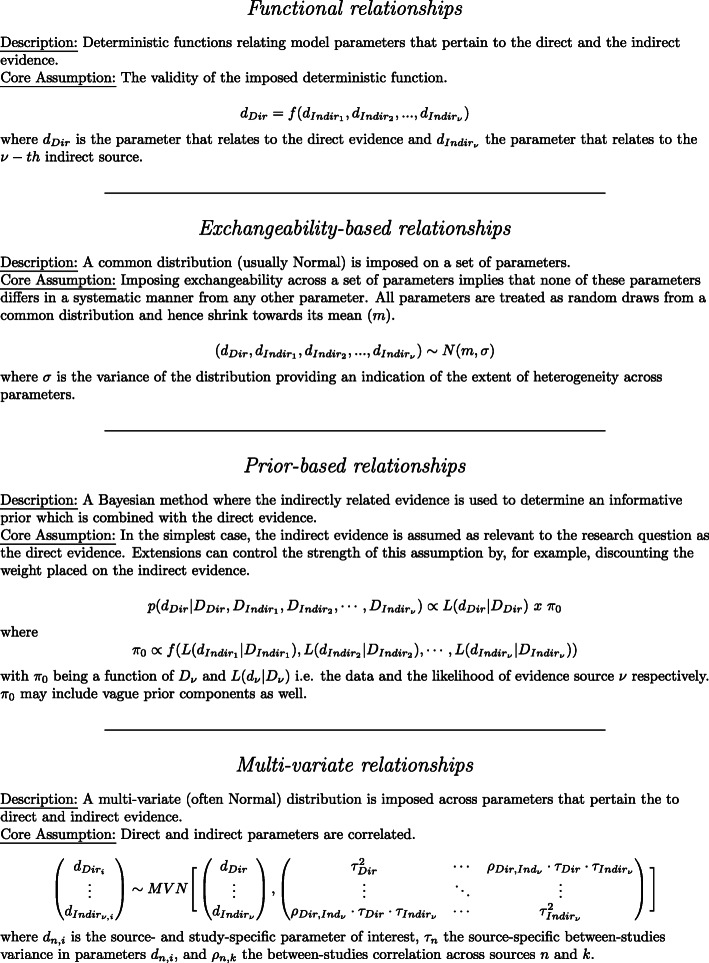
‘Core’ categories of information-sharing

The methods identified were classified according to the ‘core’ relationship facilitating information-sharing. Four ‘core‘ methods were identified: 1) **functional** relationships which include deterministic functions among model parameters resulting in a reduced number of parameters that need to be estimated; 2) **exchangeability-based** relationships which assume that a set of parameters are drawn from a common distribution that allows them to be shrunk towards its mean; 3) **prior-based** relationships which employ a Bayesian framework to ‘load’ the indirect evidence in prior distributions and 4) **multi-variate** methods which assume that model parameters are correlated and enable information-sharing through the covariance structure. Figure 3 provides a description of the main assumption and mathematical relationship imposed by each ‘core’ method.

Table 1 classifies papers according to the ‘core’ method used and the PICOS dimension of indirectness. It shows that some ‘core’ relationships are preferred when information is shared across specific PICOS dimensions. For instance, most of the identified papers sharing information across interventions either use functional or exchangeability-based relationships, and no example using priors was found. Also, papers that use multivariate relationships, do so to share information across related outcomes, not across populations or study-designs. This may be partly because the information required to implement multivariate methods for multiple populations or study-designs is usually unavailable in the literature. For instance, to synthesise evidence on multiple populations using multivariate methods, we would need studies that enrol all relevant populations and report separately for each, and such information is rarely provided.

#### Functional relationships

The simplest functional relationship is lumping (i.e. common effects) where all data points inform a single parameter independently of whether the evidence is direct or indirect. Examples include pooling RTEs across time-points [32], (sub-)populations [14, 33], or interventions of the same treatment class [30, 32, 35, 36], as well as pooling between-trial heterogeneity parameters [36, 39, 40, 43] or meta-regression slopes [34, 38, 41].

Another type of functional relationship is a constraint where a strict inequality is imposed among parameters. In a Bayesian framework, information-sharing is facilitated by preventing simulation samples that do not conform to the specified constraint. Such methods have been used to relate RTEs across dosages, expressing that higher dosages are expected to exhibit larger RTE [35, 44], describe structurally-related outcomes [57], and specify second-order consistency equations that impose a triangle inequality on the comparison-specific between-trial variances [39, 40].

Meta-regression-type methods have also been suggested. In the examples found, the relationships were usually linear -on the modelling scale- with one RTE component independent and another RTE component dependent on a particular study characteristic. The most common example in this category is bias-adjustment methods, primarily used to synthesise studies of different designs. Bias-adjustment methods broadly fall into two categories: general frameworks that adjust the RTE for biases affecting internal and external validity provided that the extent of bias can be either estimated from empirical evidence or elicited from experts [91, 92, 98, 99], and approaches that adjust for bias due to particular study-level characteristics (considered proxies for study quality such as their size [42, 50, 94–96], publication year [97], or risk-of-bias [90, 93]). Meta-regression-type relationships have also been used for complex interventions. In their simplest form, they model the RTE of a complex intervention as the sum of RTEs of its treatment components [30, 36, 47, 48]. More sophisticated approaches allow for synergistic or antagonistic relationships by suggesting functions that also contain treatment interaction RTE components [37]. Other applications include approaches that model the RTEs for two survival outcomes (e.g. time-to-mortality and time-to-progression) by assuming that they only differ by a constant component which is invariant across treatment comparisons [31], models that assume a linear relationship between dosage and RTE [35, 46], methods for baseline-risk adjustment [34], and models that relate the relative effects of populations subgroups of differing disease severity [33].

Finally, more complex, non-linear, relationships have also been presented in the literature, namely those enabling the synthesis of RTEs across a range of dosages using the Emax model [51–53] commonly employed in pharmacokinetics or other non-linear models [35] and those enabling information-sharing across follow-up periods [61, 79].

#### Exchangeability-based relationships

The simplest exchangeability-based relationship uses a random effect to relate a set of parameters; in this way accounting for heterogeneity without explicitly modelling its source(s). The random effect assumes that all parameters are drawn from a distribution, implying that individual parameters are shrunken towards the random effect mean; this can happen to a greater or lesser extent, depending on the precision and discrepancy of each individual estimate in relation to the random effect mean. Examples of parameters to which random-effects have been applied include: comparison-specific meta-regression slopes [34, 38, 41, 42, 50], comparison-specific between-trial variances [39, 40], and study-specific baseline-risks [34, 54].

Random-walks are another form of exchangeability relationship. They assume that data points which are more similar with respect to a particular characteristic are expected to exhibit more similar RTEs. Examples include approaches assuming that the RTE of a particular dosage or follow-up period is drawn from a distribution centred around the RTE of its adjacently lower or higher dosage [35] or follow-up period [45, 61].

**Fig. 4:**
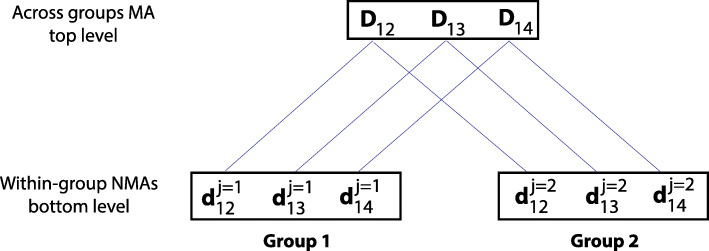
An illustration of a multi-level model

Multi-level models also use exchangeability, but apply it to the hierarchical/clustered structure of the available data. As such, exchangeability is applied at a first level within specific groups of parameters (i.e. multiple random effects are applied, each within groups of RTEs from studies showing a particular characteristic) and at a second level across the group-specific hyper-parameters. This is shown in Fig. 4, where in the bottom level, studies are categorised according to a characteristic and a different random effect is imposed within every category, producing group-specific basic parameters and heterogeneities. Subsequently, in the top-level, exchangeability is also assumed across the group-specific basic parameters which are shrunk towards an overall, global, group-independent, hyper-mean. Examples include ‘class-effect’ models where, on top of the classical Random-Effects (RE) NMA models, the basic parameters of treatments that function through the same mechanism are assumed to be drawn from a common distribution with an overall ‘class’ mean and an across-treatments, within-class, heterogeneity [32, 33, 35, 44, 46, 49]. Class-effect approaches have also been imposed across comparison-specific meta-regression slopes [38, 50]. Multi-level models have been suggested to combine adult and paediatric evidence [55], RTEs measured at different time-points [30], and studies of different designs [85, 86, 88, 100].

#### Prior-based relationships

Direct and indirect evidence can also be combined through the use of prior distributions. The process usually consists of two-steps where initially the indirect evidence is analysed and subsequently the resulting distribution is used as a prior in the analysis of the direct evidence. Of note is that this approach is mathematically equivalent to lumping, which was described under functional relationships. Examples include the combination of adult and paediatric evidence [55] or randomised and non-randomised evidence [85–89]. The prior can additionally be adjusted for bias or its precision decreased [85]. Alternative ways to define the prior include the use of meta-epidemiological evidence or expert elicitation. The former has been used primarily for bias-adjustment [90], whilst both the former [24, 107, 108] and the latter [110] have been used to define prior a distribution for the between-trials heterogeneity.

More nuanced prior-based approaches such as mixtures of priors have also been used. Here, the informative prior (distribution representing the indirect evidence) is not used at face value, but instead mixed with a vague prior according to weights that may be specified by the analyst or estimated within the synthesis model. The resulting informative prior is typically heavy-tailed, and allows for *‘adaptive’* information-sharing whereby information-sharing is stronger when the direct and indirect evidence are in agreement and weaker when they conflict [56]. Mixtures of priors have been used to combine evidence on RTE and between-studies heterogeneity across adults and children [56] and to analyse the study-specific baseline parameters from studies that enrol populations with different baseline risks [34]. The use of mixtures of priors has also been discussed for the synthesis of randomised and non-randomised evidence [85].

Finally, a flexible method that has been proposed is the power-prior [111]. In this method, the likelihood of the indirect evidence is raised to a power scalar 0≤*a*≤1 which reflects the perceived similarity between the two sources of evidence. When *a*=1 the results are equivalent to ‘lumping’ and when *a*=0 results are identical to ‘splitting’. The power parameter, *a*, needs to be specified, and it has been proposed to be elicited [112] or varied in sensitivity analysis [113]. Power priors have been used to combine observational and randomised evidence [101] and for the synthesis of adult and paediatric evidence [55].

#### Multivariate relationships

Multi-variate relationships have primarily been used to share information across multiple outcomes. Multivariate meta-analysis correlates the various outcomes and may separate within- and between-studies correlations [73]. At the within-study level, the study-specific correlations arise due to differences among the included patients and indicate how the outcomes co-vary across individuals within the study. For example, patients who, due to a baseline characteristic that makes their disease more severe, show high values for outcome A, are also more likely to yield high values for outcome B. At the between-studies level, correlations arise mainly due to study-level differences such as the distribution of the patient-level characteristics across studies. For instance, studies that enrol more severe cases and therefore may show high values for the mean of outcome A, are also more likely to result in high values for the mean of outcome B, whilst studies enrolling less severe cases may show lower mean values for both outcomes. These models can potentially produce more precise estimates [75] and mitigate outcome reporting bias [103, 104].

Multivariate methods have been developed to consider two [74, 83, 84], three or more correlated outcomes [26, 78], accommodate the simultaneous analyses of multiple treatments [63, 68, 80], and assess the relationship between surrogate and final outcomes [65, 67]. Given that within-trial correlations are commonly not reported, authors have suggested the use of external data to inform these parameters [64] or, when external data is not available, methods that approximate the within-study co-variances [77]. Further extensions have been developed to handle missing data [70], assist the estimation of the between-studies covariance matrix when only a few studies are available [71], model the within-studies covariance structure using copulas [72], and allow modelling of heterogeneity and inconsistency using two separate variance components [69].

To accommodate cases where the within-trials correlations are unavailable and cannot be otherwise obtained, alternative methods, which require the same data as a univariate approach and do not separate within- and between-trials correlations have been suggested for MA [81, 82] and NMA [80]. Assuming that the overall correlation is not very strong, these methods perform very similarly to their counterpart, which separates the two correlations, whilst preserving their benefits against the univariate approach.

Finally, some methods only account for either the within- or the between- studies correlations. For example, to model mutually exclusive outcomes, it has been suggested to only account for the within-trials negative correlations which are induced by the competing risks structure of the data (i.e. the more patients that reach an outcome, the fewer the patients that reach another outcome) [62]. Also, other approaches have only modelled the between-studies covariance matrix to allow simultaneous synthesis of multiple outcomes [30, 31, 57, 60], accommodate outcomes reported at several follow-up periods [58, 59] and enable information-sharing across different treatment components of complex interventions [36].

## Discussion

The aim of this review was to identify and classify evidence synthesis methods that have been used to combine evidence from sources that relate directly and indirectly to a particular research question. A wide range of methods have been developed to share information between populations, treatments, outcomes and study-designs. We found that across the breadth of methods identified, four ‘core’ relationships are used to facilitate information-sharing. These are functional, exchangeability-based, prior-based, and multivariate relationships and are illustrated in Fig. 3.

This review highlights the breadth of methodological options that can facilitate information-sharing. Although, typically, particular relationships are used preferentially to share information on specific information-sharing contexts, it is likely that several methods are applicable and analysts would need to choose which method is more appropriate. This paper highlights that appropriate considerations need to be made when choosing ‘core’ relationships and methods because choices are likely to influence the degree of information-sharing. Specifically, method selection may be informed by the following considerations; the first is the plausibility of the assumptions imposed by the methods in the context of interest. By classifying methods according to the ‘core‘ relationship that enables information-sharing, we hope to facilitate a clearer discussion about the plausibility of these assumptions in the decision context of interest.

The second is the degree of information-sharing that is imposed between direct and indirect evidence. Within the literature, there is limited exploration of how much different methods borrow-strength from indirect evidence, though for multivariate methods, it has been noted that information-sharing is ‘usually modest’ [26, 66] and, sometimes, instead of ‘borrowing-strength’, multi-variate methods may end up ‘borrowing-weakness’ [114]. The few studies that have assessed the degree of information-sharing typically consider only the degree of precision gains [115] rather than also examining how the point estimate - which is also important for decision making - changes. Further research to understand the extent to which different methods share information is warranted.

Finally, decision-makers may be interested in exploring different levels of information-sharing. One way to do that is by using prior-based methods that allow some control on the degree of information-sharing. For instance, an informative prior may use either the posterior distribution of the mean, or the predictive distribution of the indirect evidence. The former is equivalent to lumping, whilst the latter imposes less information-sharing. Similarly, mixture priors can regulate the weight that is placed on the informative component, and power-priors allow a range of values to be used for *α* which determines the extent of information-sharing.

Whilst our literature search was systematic in its methods and conduct, it is unlikely to have been fully comprehensive. The use of citation-mining techniques, while efficient and necessary for this search, may have missed relevant methods. This is because the sensitivity of citation-mining methods depends on the existence and identification of seminal papers [22] and on papers citing the most impactful references [116]. Due to time lags in citations, this technique may not capture recent developments within a field [117]. We have also excluded methods developed outside of health research and did not specifically target the grey literature. Since the search was conducted, we found in the grey literature a relevant method using multivariate methods to simultaneously synthesise the relative effects of patients treated at different lines of treatment [118]. However, we do not believe that the conclusions of a comprehensive search (had it been possible) would differ from those in this paper, namely regarding the core relationships identified, and the focus of sharing being on sharing across outcomes and treatments. We would also like to highlight that it would be important that further research considers methods developed for different purposes that could be applied for information sharing. One example is commensurate priors which have been used to combine individual-patient data and aggregate-level evidence [119].

This paper is the first to summarise and categorise the existing literature by classifying methods according to the ‘core’ assumption that they use to facilitate information-sharing. Further research could explore the following questions: first, how can we determine whether indirect evidence is relevant? Second, how can the appropriateness of each information-sharing method be assessed for the synthesis problem at hand? Finally, can the extent of information-sharing be quantified to assist transparent decision-making?

## Conclusions

We conclude that a plethora of methods has been used to facilitate information-sharing. These can be categorised according to the main assumption they impose into functional, exchangeability-based, prior-based, and multivariate relationships. Despite the wide range of available methods, these are often used preferentially without ensuring that all options have been explored. Given that methods may differ in the degree of information-sharing they impose, the implication is that the chosen method may impose stronger or weaker information-sharing that what is considered appropriate by policy-makers. Further research should investigate ways of judging the appropriateness of the degree of information-sharing imposed by each method, and assess the impact of using different methods on decisions.

## Supplementary Information

**Additional file 1** Search strategy. A description of the inclusion and exclusion criteria of the search as well as the number of citations for each one of the seminal papers and the number of times it has been cited.

**Additional file 2** PRISMA checklist for systematic reviews. The PRISMA checklist for systematic reviews, indicating the page at which each characteristic of the review is described.

**Additional file 3** Brief summary of each included study. Multi-page tables including a brief description of how each of the included papers shared information between direct and indirect information, on which PICOS dimension and its main characteristics.

## Data Availability

The list of included studies in the literature review is available online in the following https://www.zotero.org/groups/2360368/citation-mining_included-studies. Declarations

## Abbreviations

DAM: Decision analytic model
EMA: European medicines agency
FDA: U.S. food and drugs administration
HTA: Health technology assessment
MA: Meta-analysis
NHS: National health service
NICE: National Institute for Health and Care Excellence
NMA: Network meta-analysis
QoL: Quality of life
RE: Random-effects
RTE: Relative treatment effect
TA: Technology appraisal

## References

[1] World Health Organization. WHO HTA Definition (EB 134/30). 2018. http://www.who.int/health-technology-assessment/about/Defining/en/. Accessed 1 Apr 2021.

[2] Claxton K, Martin S, Soares M, Rice N, Spackman E, Hinde S, Devlin N, Smith PC, Sculpher M (2015). Methods for the estimation of the National Institute for Health and Care Excellence cost-effectiveness threshold. Health Technol Assess (Winchester, England).

[3] Drummond MF, Sculpher MJ, Claxton K, Stoddart GL, Torrance GW (2015). Methods for the Economic Evaluation of Health Care Programmes.

[4] Briggs A, Claxton K, Sculpher MJ (2006). Decision Modelling for Health Economic Evaluation.

[5] Centre for Reviews and Dissemination. Systematic reviews. CRD‘s guidance for undertaking reviews in health care. Centre for Reviews and Dissemination. 2009. https://www.york.ac.uk/media/crd/Systematic_Reviews.pdf.

[6] DerSimonian R, Laird N (1986). Meta-analysis in clinical trials. Control Clin Trials.

[7] Lu G, Ades AE (2004). Combination of direct and indirect evidence in mixed treatment comparisons. Stat Med.

[8] Ades AE, Sutton AJ (2006). Multiparameter evidence synthesis in epidemiology and medical decision-making: current approaches. J R Stat Soc Ser A (Stat Soc).

[9] Sweeting MJ, Sutton AJ, Lambert PC (2004). What to add to nothing? use and avoidance of continuity corrections in meta-analysis of sparse data. Stat Med.

[10] IntHout J, Ioannidis JPA, Borm GF, Goeman JJ (2015). Small studies are more heterogeneous than large ones: a meta-meta-analysis. J Clin Epidemiol.

[11] Food and Drug Administrations, Center for Devices and Radiological Health, Center for Biologics Evaluation and Research. Leveraging Existing Clinical Data for Extrapolation to Pediatric Uses of Medical Devices. 2016. https://www.fda.gov/regulatory-information/search-fda-guidance-documents/leveraging-existing-clinical-data-extrapolation-pediatric-uses-medical-devices.

[12] European Medicines Agency. Reflection paper on the use of extrapolation in the development of medicines for paediatrics. 2016. https://www.ema.europa.eu/en/documents/scientific-guideline/adopted-reflection-paper-use-extrapolation-development-medicines-paediatrics-revision-1_en.pdf.

[13] Ohlssen D, Price KL, Amy Xia H, Hong H, Kerman J, Fu H, Quartey G, Heilmann CR, Ma H, Carlin BP (2014). Guidance on the implementation and reporting of a drug safety bayesian network meta-analysis. Pharm Stat.

[14] Duarte A, Mebrahtu T, Goncalves PS, Harden M, Murphy R, Palmer S, Woolacott N, Rodgers M, Rothery C (2017). Adalimumab RC, etanercept and ustekinumab for treating plaque psoriasis in children and young people: systematic review and economic evaluation. Health Technol Assess.

[15] Faria R, Woods B, Griffin S, Palmer S, Sculpher M, Ryder SD (2016). Prevention of progression to cirrhosis in hepatitis c with fibrosis: effectiveness and cost effectiveness of sequential therapy with new direct-acting anti-virals. Aliment Pharmacol Ther.

[16] Corbett M, Soares M, Jhuti G, Rice S, Spackman E, Sideris E, Moe-Byrne T, Fox D, Marzo-Ortega H, Kay L, Woolacott N, Palmer S (2016). Tumour necrosis factor- *a* inhibitors for ankylosing spondylitis and non-radiographic axial spondyloarthritis: a systematic review and economic evaluation. Health Technol Assess.

[17] McDaid C, Griffin S, Weatherly H, Duree K, van der Burgt M, van Hout S, Akers J, Davies RJ, Sculpher M, Westwood M (2009). Continuous positive airway pressure devices for the treatment of obstructive sleep apnoea-hypopnoea syndrome: a systematic review and economic analysis. Health Technol Assess.

[18] Burch J, Paulden M, Conti S, Stock C, Corbett M, Welton NJ, Ades AE, Sutton A, Cooper N, Elliot AJ, Nicholson K, Duffy S, McKenna C, Stewart L, Westwood M, Palmer S (2008). Antiviral drugs for the treatment of influenza: A systematic review and economic evaluation. Health Technol Assess.

[19] Riemsma R, Lhachimi SK, Armstrong N, van Asselt ADI, Allen A, Manning N, Harker J, Tushabe DA, Severens JL, Kleijnen J. Roflumilast for the management of severe chronic obstructive pulmonary disease: A single technology appraisal. York: Kleijnen Systematic Reviews Ltd: 2017.

[20] Higgins JPT, Thomas J, Chandler J, Cumpston M, Li T, Page MJ, Welch VA. Cochrane Handbook for Systematic Reviews of Interventions version 6.2 (updated February 2021). Cochrane; 2021. Available from www.training.cochrane.org/handbook.

[21] Grandage K, Slawson D, Shaughnessy AF (2002). Site-ation pearl growing: methods and librarianship history and theory. J Med Libr Assoc.

[22] Badampudi D, Wohlin C, Petersen K (2015). Experiences from using snowballing and database searches in systematic literature studies. Proceedings of the 19th International Conference on Evaluation and Assessment in Software Engineering EASE ’15.

[23] Verde PE, Ohmann C (2015). Combining randomized and non-randomized evidence in clinical research: a review of methods and applications. Res Synth Methods.

[24] Higgins JPT, Whitehead A (1996). Borrowing strength from external trials in a meta-analysis. Stat Med.

[25] Ades AE, Sculpher M, Sutton A, Abrams K, Cooper N, Welton N, Lu G (2006). Bayesian methods for evidence synthesis in cost-effectiveness analysis. PharmacoEconomics.

[26] Jackson D, Riley R, White IR (2011). Multivariate meta-analysis: Potential and promise. Stat Med.

[27] Efthimiou O, Debray TPA, van Valkenhoef G, Trelle S, Panayidou K, Moons KGM, Reitsma JB, Shang A, Salanti G, on behalf of GetReal Methods Review Group (2016). Getreal in network meta-analysis: a review of the methodology. Res Synth Methods.

[28] Hobbs BP, Carlin BP, Mandrekar SJ, Sargent DJ (2011). Hierarchical commensurate and power prior models for adaptive incorporation of historical information in clinical trials. Biom.

[29] Schmidli H, Gsteiger S, Roychoudhury S, O’Hagan A, Spiegelhalter D, Neuenschwander B (2014). Robust meta-analytic-predictive priors in clinical trials with historical control information. Biom.

[30] Madan J, Chen Y-F, Aveyard P, Wang D, Yahaya I, Munafo M, Bauld L, Welton N (2014). Synthesis of evidence on heterogeneous interventions with multiple outcomes recorded over multiple follow-up times reported inconsistently: a smoking cessation case-study. J R Stat Soc Ser A (Stat Soc).

[31] Welton NJ, Willis SR, Ades AE (2010). Synthesis of survival and disease progression outcomes for health technology assessment of cancer therapies. Res Synth Methods.

[32] Dakin HA, Welton NJ, Ades AE, Collins S, Orme M, Kelly S (2011). Mixed treatment comparison of repeated measurements of a continuous endpoint: an example using topical treatments for primary open-angle glaucoma and ocular hypertension. Stat Med.

[33] Soares MO, Dumville JC, Ades AE, Welton NJ (2014). Treatment comparisons for decision making: facing the problems of sparse and few data. J R Stat Soc Ser A (Stat Soc).

[34] Achana FA, Cooper NJ, Dias S, Lu G, Rice SJ, Kendrick D, Sutton AJ (2013). Extending methods for investigating the relationship between treatment effect and baseline risk from pairwise meta-analysis to network meta-analysis. Stat Med.

[35] Del Giovane C, Vacchi L, Mavridis D, Filippini G, Salanti G (2013). Network meta-analysis models to account for variability in treatment definitions: application to dose effects. Stat Med.

[36] Nixon RM, Bansback N, Brennan A (2007). Using mixed treatment comparisons and meta-regression to perform indirect comparisons to estimate the efficacy of biologic treatments in rheumatoid arthritis. Stat Med.

[37] Welton NJ, Caldwell DM, Adamopoulos E, Vedhara K (2009). Mixed treatment comparison meta-analysis of complex interventions: psychological interventions in coronary heart disease. Am J Epidemiol.

[38] Cooper NJ, Sutton AJ, Morris D, Ades AE, Welton NJ (2009). Addressing between-study heterogeneity and inconsistency in mixed treatment comparisons: Application to stroke prevention treatments in individuals with non-rheumatic atrial fibrillation. Stat Med.

[39] Thorlund K, Thabane L, Mills EJ (2013). Modelling heterogeneity variances in multiple treatment comparison meta-analysis. are informative priors the better solution?. BMC Med Res Methodol.

[40] Lu G, Ades A (2009). Modeling between-trial variance structure in mixed treatment comparisons. Biostat.

[41] Dias S, Sutton A, Welton N, Ades A. Nice dsu technical support document 3: Heterogeneity: Subgroups, meta-regression, bias and bias-adjustment. 2011. last updated April 2012; available from http://www.nicedsu.org.uk. 27905717

[42] Chaimani A, Salanti G (2012). Using network meta-analysis to evaluate the existence of small-study effects in a network of interventions. Res Synth Methods.

[43] Dias S, Welton N, Sutton A, Ades A. Nice dsu technical support document 2: A generalised linear modelling framework for pairwise and network meta-analysis of randomised controlled trials. 2011; TSD2. 27466657

[44] Owen RK, Tincello DG, Keith RA (2015). Network meta-analysis: development of a three-level hierarchical modeling approach incorporating dose-related constraints. Value Health.

[45] da Costa BR, Reichenbach S, Keller N, Nartey L, Wandel S, Juni P, Trelle S (2017). Effectiveness of non-steroidal anti-inflammatory drugs for the treatment of pain in knee and hip osteoarthritis: a network meta-analysis. Lancet.

[46] Warren FC, Abrams KR, Sutton AJ (2014). Hierarchical network meta-analysis models to address sparsity of events and differing treatment classifications with regard to adverse outcomes. Stat Med.

[47] Mills EJ, Thorlund K, Ioannidis JPA (2012). Calculating additive treatment effects from multiple randomized trials provides useful estimates of combination therapies. J Clin Epidemiol.

[48] Melendez-Torres GJ, Bonell C, Thomas J (2015). Emergent approaches to the meta-analysis of multiple heterogeneous complex interventions. BMC Med Res Methodol.

[49] Dominici F, Parmigiani G, Wolpert RL, Hasselblad V (1999). Meta-analysis of migraine headache treatments: Combining information from heterogeneous designs. J Am Stat Assoc.

[50] Moreno SG, Sutton AJ, Ades AE, Cooper NJ, Abrams KR (2011). Adjusting for publication biases across similar interventions performed well when compared with gold standard data. J Clin Epidemiol.

[51] Mawdsley D, Bennetts M, Dias S, Boucher M, Welton NJ (2016). Model-based network meta-analysis: A framework for evidence synthesis of clinical trial data. CPT Pharmacometrics Syst Pharmacol.

[52] Wu J, Banerjee A, Jin B, Menon SM, Martin SW, Heatherington AC (2018). Clinical dose-response for a broad set of biological products: A model-based meta-analysis. Stat Methods Med Res.

[53] Langford O, Aronson JK, van Valkenhoef G, Stevens RJ (2018). Methods for meta-analysis of pharmacodynamic dose-response data with application to multi-arm studies of alogliptin. Stat Methods Med Res.

[54] Dias S, Welton N, Sutton A, Ades A. NICE DSU Technical Support Document 1: Introduction to evidence synthesis for decision making. 2011. last updated April 2012; available from http://www.nicedsu.org.uk. 27905715

[55] Gamalo-Siebers M, Savic J, Basu C, Zhao X, Gopalakrishnan M, Gao A, Song G, Baygani S, Thompson L, Xia HA, Price K, Tiwari R, Carlin BP (2017). Statistical modeling for Bayesian extrapolation of adult clinical trial information in pediatric drug evaluation. Pharm Stat.

[56] Roever C, Wandel S, Friede T (2019). Model averaging for robust extrapolation in evidence synthesis. Stat Med.

[57] Welton NJ, Cooper NJ, Ades AE, Lu G, Sutton AJ (2008). Mixed treatment comparison with multiple outcomes reported inconsistently across trials: evaluation of antivirals for treatment of influenza a and b. Stat Med.

[58] Jackson D, Rollins K, Coughlin P (2014). A multivariate model for the meta-analysis of study level survival data at multiple times. Res Synth Methods.

[59] Musekiwa A, Manda SOM, Mwambi HG, Chen D-G. Meta-Analysis of Effect Sizes Reported at Multiple Time Points Using General Linear Mixed Model. PLOS ONE. 2016; 11(10). 10.1371/journal.pone.0164898 PMC5087886 27798661

[60] Hong H, Chu H, Zhang J, Carlin BP (2016). A bayesian missing data framework for generalized multiple outcome mixed treatment comparisons. Res Synth Methods.

[61] Lu G, Ades AE, Sutton AJ, Cooper NJ, Briggs AH, Caldwell DM (2007). Meta-analysis of mixed treatment comparisons at multiple follow-up times. Stat Med.

[62] Ades AE, Mavranezouli I, Dias S, Welton NJ, Whittington C, Kendall T (2010). Network meta-analysis with competing risk outcomes. Value Health.

[63] Achana FA, Cooper NJ, Bujkiewicz S, Hubbard SJ, Kendrick D, Jones DR, Sutton AJ (2014). Network meta-analysis of multiple outcome measures accounting for borrowing of information across outcomes. BMC Med Res Methodol.

[64] Bujkiewicz S, Thompson JR, Sutton AJ, Cooper NJ, Harrison MJ, Symmons DPM, Abrams KR (2014). Use of bayesian multivariate meta-analysis to estimate the haq for mapping onto the eq-5d questionnaire in rheumatoid arthritis. Value in Health.

[65] Bujkiewicz S, Thompson JR, Riley RD, Abrams KR (2016). Bayesian meta-analytical methods to incorporate multiple surrogate endpoints in drug development process. Stat Med.

[66] Copas JB, Jackson D, White IR, Riley RD (2018). The role of secondary outcomes in multivariate meta-analysis. J R Stat Soc Ser C Appl Stat.

[67] Daniels MJ, Hughes MD (1997). Meta-analysis for the evaluation of potential surrogate markers. Stat Med.

[68] Efthimiou O, Mavridis D, Cipriani A, Leucht S, Bagos P, Salanti G (2014). An approach for modelling multiple correlated outcomes in a network of interventions using odds ratios. Stat Med.

[69] Jackson D, Bujkiewicz S, Law M, Riley RD, White IR (2018). A matrix-based method of moments for fitting multivariate network meta-analysis models with multiple outcomes and random inconsistency effects. Biometrics.

[70] Jackson D, White IR, Riley RD (2013). A matrix-based method of moments for fitting the multivariate random effects model for meta-analysis and meta-regression. Biom J.

[71] Jackson D, Riley RD (2014). A refined method for multivariate meta-analysis and meta-regression. Stat Med.

[72] Liu Y, DeSantis SM, Chen Y (2018). Bayesian mixed treatment comparisons meta-analysis for correlated outcomes subject to reporting bias. J R Stat Soc Ser C Appl Stat.

[73] Mavridis D, Salanti G (2013). A practical introduction to multivariate meta-analysis. Stat Methods Med Res.

[74] Nam I, Mengersen K, Garthwaite P (2003). Multivariate meta-analysis. Stat Med.

[75] Riley RD, Abrams KR, Lambert PC, Sutton AJ, Thompson JR (2007). An evaluation of bivariate random-effects meta-analysis for the joint synthesis of two correlated outcomes. Stat Med.

[76] Tan SH, Abrams KR, Bujkiewicz S (2018). Bayesian Multiparameter Evidence Synthesis to Inform Decision Making: A Case Study in Metastatic Hormone-Refractory Prostate Cancer. Med Decis Making.

[77] Wei Y, Higgins JPT (2013). Estimating within-study covariances in multivariate meta-analysis with multiple outcomes. Stat Med.

[78] Wei Y, Higgins JPT (2013). Bayesian multivariate meta-analysis with multiple outcomes. Stat Med.

[79] Ding Y, Fu H (2013). Bayesian indirect and mixed treatment comparisons across longitudinal time points. Stat Med.

[80] Efthimiou O, Mavridis D, Riley RD, Cipriani A, Salanti G (2015). Joint synthesis of multiple correlated outcomes in networks of interventions. Biostatistics.

[81] Hong C, Riley RD, Chen Y (2018). An improved method for bivariate meta-analysis when within-study correlations are unknown. Res Synth Methods.

[82] Riley RD, Thompson JR, Abrams KR (2008). An alternative model for bivariate random-effects meta-analysis when the within-study correlations are unknown. Biostatistics.

[83] van Houwelingen H, Zwinderman K, Stijnen T (1993). A bivariate approach to meta-analysis. Stat Med.

[84] van Houwelingen H, Arends L, Stijnen T (2002). Advanced methods in meta-analysis: multivariate approach and meta-regression. Stat Med.

[85] Efthimiou O, Mavridis D, Debray TPA, Samara M, Belger M, Siontis GCM, Leucht S, Salanti G, on behalf of GetReal Work P (2017). Combining randomized and non-randomized evidence in network meta-analysis. Stat Med.

[86] Schmitz S, Adams R, Walsh C (2013). Incorporating data from various trial designs into a mixed treatment comparison model. Stat Med.

[87] Mak A, Cheung MW, Ho RC, Cheak AA, Lau CS (2009). Bisphosphonates and atrial fibrillation: Bayesian meta-analyses of randomized controlled trials and observational studies. BMC Musculoskelet Disord.

[88] McCarron CE, Pullenayegum EM, Thabane L, Goeree R, Tarride JE (2010). The importance of adjusting for potential confounders in Bayesian hierarchical models synthesising evidence from randomised and non-randomised studies: an application comparing treatments for abdominal aortic aneurysms. BMC Med Res Methodol.

[89] McCarron CE, Pullenayegum EM, Thabane L, Goeree R, Tarride JE (2011). Bayesian hierarchical models combining different study types and adjusting for covariate imbalances: a simulation study to assess model performance. PLoS One.

[90] Welton NJ, Ades AE, Carlin JB, Altman DG, Sterne JAC (2009). Models for Potentially Biased Evidence in Meta-Analysis Using Empirically Based Priors. J R Stat Soc Ser A (Stat Soc).

[91] Turner RM, Spiegelhalter DJ, Smith GCS, Thompson SG (2009). Bias modelling in evidence synthesis. J R Stat Soc Ser A (Stat Soc).

[92] Spiegelhalter DJ, Best NG (2003). Bayesian approaches to multiple sources of evidence and uncertainty in complex cost-effectiveness modelling. Stat Med.

[93] Dias S, Welton NJ, Marinho VCC, Salanti G, Higgins JPT, Ades AE (2010). Estimation and adjustment of bias in randomized evidence by using mixed treatment comparison meta-analysis. J R Stat Soc Ser A (Stat Soc).

[94] Trinquart L, Chatellier G, Ravaud P (2012). Adjustment for reporting bias in network meta-analysis of antidepressant trials. BMC Med Res Methodol.

[95] Mavridis D, Sutton A, Cipriani A, Salanti G (2013). A fully bayesian application of the copas selection model for publication bias extended to network meta-analysis. Stat Med.

[96] Salanti G, Dias S, Welton NJ, Ades AE, Golfinopoulos V, Kyrgiou M, Mauri D, Ioannidis JP (2010). Evaluating novel agent effects in multiple-treatments meta-regression. Stat Med.

[97] Salanti G, Marinho V, Higgins JP (2009). A case study of multiple-treatments meta-analysis demonstrates that covariates should be considered. J Clin Epidemiol.

[98] Eddy DM, Hasselblad V, Shachter R (1990). An introduction to a bayesian method for meta-analysis: The confidence profile method. Med Decis Making.

[99] Wolpert RL, Kerrie LM (2004). Adjusted likelihoods for synthesizing empirical evidence from studies that differ in quality and design: Effects of environmental tobacco smoke. Stat Sci.

[100] Prevost T, Abrams K, Jones D (2000). Hierarchical models in generalized synthesis of evidence: an example based on studies of breast cancer screening. Stat Med.

[101] Rietbergen C. Quantitative evidence synthesis with power priors. PhD thesis, Utrecht University. 2016. http://dspace.library.uu.nl/handle/1874/329030.

[102] Ades AE, Welton NJ, Caldwell D, Price M, Goubar A, Lu G (2008). Multiparameter evidence synthesis in epidemiology and medical decision-making. J Health Serv Res Policy.

[103] Hwang H, DeSantis SM (2018). Multivariate network meta-analysis to mitigate the effects of outcome reporting bias. Stat Med.

[104] Kirkham JJ, Riley RD, Williamson PR (2012). A multivariate meta-analysis approach for reducing the impact of outcome reporting bias in systematic reviews. Stat Med.

[105] Lu G, Kounali D, Ades AE (2014). Simultaneous Multioutcome Synthesis and Mapping of Treatment Effects to a Common Scale. Value Health.

[106] Riley RD, Abrams KR, Sutton AJ, Lambert PC, Thompson JR (2007). Bivariate random-effects meta-analysis and the estimation of between-study correlation. BMC Med Res Methodol.

[107] Turner RM, Jackson D, Wei Y, Thompson SG, Higgins JPT (2015). Predictive distributions for between-study heterogeneity and simple methods for their application in Bayesian meta-analysis. Stat Med.

[108] Pullenayegum EM (2011). An informed reference prior for between-study heterogeneity in meta-analyses of binary outcomes. Stat Med.

[109] Rhodes KM, Turner RM, Higgins JPT (2015). Predictive distributions were developed for the extent of heterogeneity in meta-analyses of continuous outcome data. J Clin Epidemiol.

[110] Ren S, Oakley JE, Stevens JW (2018). Incorporating Genuine Prior Information about Between-Study Heterogeneity in Random Effects Pairwise and Network Meta-analyses. Med Decis Making.

[111] Ibrahim JG, Chen M-H (2000). Power prior distributions for regression models. Statist Sci.

[112] Rietbergen C, Groenwold RHH, Hoijtink HJA, Moons KGM, Klugkist I (2016). Expert elicitation of study weights for bayesian analysis and meta-analysis. J Mixed Methods Res.

[113] Spiegelhalter DJ, Abrams R, Myles JP. Bayesian approaches to clinical trials and health-care evaluation.Wiley; 2004.

[114] Bujkiewicz S, Thompson JR, Sutton AJ, Cooper NJ, Harrison MJ, Symmons DPM, Abrams KR (2013). Multivariate meta-analysis of mixed outcomes: a bayesian approach. Stat Med.

[115] Jackson D, White IR, Price M, Copas J, Riley RD (2017). Borrowing of strength and study weights in multivariate and network meta-analysis. Stat Methods Med Res.

[116] Sylvia MJ (1998). Citation analysis as an unobtrusive method for journal collection evaluation using psychology student research bibliographies. Collect Build.

[117] Johnson P (2014). Fundamentals of Collection Development and Management: Third Edition.

[118] Abrams K, Bujkiewicz S, Dequen P, Jenkins D, Martina R. WP1: Deliverable 1.5 (Case Study Review: Rheumatoid Arthritis) - GetReal - Project No. 115546. 2016. https://www.imi-getreal.eu/Portals/1/Documents/01%20deliverables/Deliverable%20Report%20D1.5_Rheumatoid%20Arthritis_websiteversion.pdf.

[119] Hong H, Fu H, Carlin BP. Power and commensurate priors for synthesizing aggregate and individual patient level data in network meta-analysis. J R Stat Soc Ser C (Appl Stat). 2018.

