# Supplementary material for: Classifying information-sharing methods
Source: BMC Med Res Methodol. 2021 May 22;21:107. doi: 10.1186/s12874-021-01292-z (PMC8140466; doi:10.1186/s12874-021-01292-z)
Supplement: Supplementary file 1 — Additional file 1 Search strategy. A description of the inclusion and exclusion criteria of the search as well as the number of citations for each one of the seminal papers and the number of times it has been cited. [file 12874_2021_1292_MOESM1_ESM.pdf]

Additional file 1: Search strategy

Georgios F. Nikolaidis

December 2020

1 Scoping the literature

Our scoping review was used as a precursor to our systematic review with a view to informing its design and conduct. Its main aims were:

1. To clarify the working definitions and understand whether consistent terminology is used in the literature to describe methods that borrow strength.
2. To determine the range of research fields that have developed methods that borrow strength and assist the definition of appropriate and realistic inclusion and exclusion criteria in our systematic review.
3. To understand whether the most suitable way of systematically searching the literature is by using keyword-based or citation-mining methods.
4. To obtain a comprehensive list of representative seminal papers on information-sharing.
5. To conceive how the breadth of information-sharing methods could be categorised in a useful manner

To fulfil the aforementioned aims we used the citations and references of the most widely cited paper on the topic of "Borrowing of strength" (i.e. Higgins and Whitehead [4]) and conducted targeted searches in MEDLINE combing keywords on information-sharing ('borrowing of strength', 'borrow strength', 'borrow-strength', 'information sharing', 'information-sharing') with keywords on evidence synthesis ('meta-analysis', 'network meta-analysis', 'mixed treatment comparison', 'multiple treatment comparison', 'indirect comparison', 'simultaneous treatment comparison').

We found that there was no consistent terminology on the subject and although 'borrowing strength' was the term most often mentioned in relevant papers, there were still several papers that developed relevant methods and did not use that term or any other term referring to information-sharing or borrowing of strength. Furthermore, given that our review aimed to identify statistical methods, its research question could not be framed under the PICO format for which classical keyword-based methods are well suited. Therefore, we decided that a citation-mining review was the most appropriate way forward.

2 Determining the list of seminal papers

To identify our seminal papers, we used the results of our literature scoping process. Given that (Higgins and Whitehead [4]) was the most cited paper, it was directly included in our list of seminal papers. Amongst the other papers identified in the scoping process, we selected papers that not only were also widely cited, but also represented a variety of research fields. Specifically, Ades and Sutton [1] was selected to represent the field of multi-parameter evidence synthesis, Ades et al. [2] to represent the field of cost-effectiveness and policy-making, Efthimiou et al. [3] to represent the field of NMA, Mavridis and Salanti [8] to represent the field of multi-variate synthesis of multiple outcomes, and Schmidli et al. [9] to represent the field of incorporating evidence from historical controls.

The list of those 6 papers was then discussed with two external experts in evidence synthesis. It was noted that Jackson et al. [6] although not included in the list of papers cited by Higgins and Whitehead [4], was more widely cited (by n=148 papers by the end of 2018) than Mavridis and Salanti [8] (n = 47) and was perhaps a better option to represent the field of multivariate

Table 1: *'Pearls' (i.e. seminal papers) used for forwards and backwards citation-mining*

#	'Pearl'	Citations	Cited by
1	Higgins and Whitehead, 1996. *Borrowing strength from external trials in a meta-analysis*	33	309
2	Ades and Sutton 2006 *Multiparameter evidence synthesis in epidemiology and medical decision-making: current approaches*	109	82
3	Ades et al., 2006 *Bayesian methods for evidence synthesis in cost-effectiveness analysis*	79	210
4	Jackson et al., 2011 *Multivariate meta-analysis: Potential and promise*	74	148
5	Efthimiou et al., 2016 *GetReal in network meta-analysis: a review of the methodology*	193	37
6	Hobbs et al., 2011 *Hierarchical commensurate and power prior models for adaptive incorporation of historical information in clinical trials*	16	64
7	Schmidli et al., 2014 *Robust meta-analytic-predictive priors in clinical trials with historical control information*	50	42

meta-analysis. As such, the proposed substitution was implemented. Finally, another paper that had not also cited Higgins and Whitehead [4] and related to the field of the incorporation of information from historical controls was brought to our attention by the panel (Hobbs et al. [5]). Given that our uncertainty in whether this paper should substitute Schmidli et al. [9], we decided to simply add it to the list of seminal papers without making any exclusions.

Overall, 7 seminal papers made the final list and were used for our systematic review and these are shown in Table 1.

3 Citation-mining review

We developed a protocol detailing the citation-mining reviewing methods that we would use; the protocol was not registered or published. The identified papers were screened by a single reviewer (GN) against the following inclusion and exclusion criteria:

Inclusion criteria

Papers were included if they specified in mathematical notation or programming code meta-analytic (MA) or network meta-analytic (NMA) methods that combined information from comparative studies that pertained to multiple populations, interventions, outcomes, study-designs or utilised evidence from an external source such as previous meta-analyses. Examples of such models included but were not restricted to hierarchical models, dose response models, meta-regression models, power-prior models, informative and mixture prior models, multivariate meta-analytic models.

Exclusion criteria

Papers were excluded from the search if they fell in any of the categories below:

1. Methods or applications developed outside the health research field (e.g. ecology)
2. Applications of standard MA/NMA methods without any extensions or developments to accommodate the inclusion of indirect information
3. Papers that did not present a synthesis model as defined in the inclusion criteria, but instead focused on other related methodological developments. Examples include but are not limited to
    - Papers that use or develop graphical/presentational methods for MA/NMA;
    - Papers that use or develop methods intended to assess consistency of the evidence;
    - Papers that use only standard NMA methods originally described by Lu and Ades [7] (i.e. pooling evidence sets and assuming perfect exchangeability), and do not advance this as specified in the inclusion criteria;

- Reviews of the quality of the statistical methods that are used in MA/NMA; and
- Papers that, outside the fields of MA and NMA, use or develop methods to combine sources of information; for example, methods that aim to utilise evidence from historical controls in the design of future trials or fall outside the field of comparative effectiveness research.

4. Protocols for the conduct or analysis of a future study
5. The University of York could not provide access to the full-text article

Data extraction

Data extraction was conducted at the paper level by a single reviewer (GN). Where there was uncertainty about the interpretation of the statistical models agreement was reached between three reviewers (GN, BW, MS). The following characteristics where extracted:

- Year of publication
- The synthesis challenge the paper aimed to address
- The information-sharing method used
- The PICOS dimension(s) of indirectness on which information was shared
- The information-sharing 'core(s)' utilised
- The parameter on which information-sharing was imposed
- Whether the paper developed methods for MA or NMA

References

[1] Ades, A.E., Sutton, A.J.: Multiparameter evidence synthesis in epidemiology and medical decision-making: current approaches. Journal of the Royal Statistical Society: Series A (Statistics in Society) **169**(1), 5–35 (2006)

[2] Ades, A.E., Sculpher, M., Sutton, A., Abrams, K., Cooper, N., Welton, N., Lu, G.: Bayesian methods for evidence synthesis in cost-effectiveness analysis. PharmacoEconomics **24**, 1–19 (2006)

[3] Efthimiou, O., Debray, T.P.A., van Valkenhoef, G., Trelle, S., Panayidou, K., Moons, K.G.M., Reitsma, J.B., Shang, A., Salanti, G., on behalf of GetReal Methods Review Group: Getreal in network meta-analysis: a review of the methodology. Research Synthesis Methods **7**(3), 236–263 (2016)

[4] Higgins, J.P.T., Whitehead, A.: Borrowing strength from external trials in a meta-analysis. Statistics in Medicine **15**(24), 2733–2749 (1996)

[5] Hobbs, B.P., Carlin, B.P., Mandrekar, S.J., Sargent, D.J.: Hierarchical commensurate and power prior models for adaptive incorporation of historical information in clinical trials. Biometrics **67**(3), 1047–56 (2011)

[6] Jackson, D., Riley, R., White, I.R.: Multivariate meta-analysis: Potential and promise. Statistics in Medicine **30**(20), 2481–2498 (2011)

[7] Lu, G., Ades, A.E.: Combination of direct and indirect evidence in mixed treatment comparisons. Statistics in Medicine **23**(20), 3105–3124 (2004)

[8] Mavridis, D., Salanti, G.: A practical introduction to multivariate meta-analysis. Stat Methods Med Res **22**(2), 133–58 (2013)

[9] Schmidli, H., Gsteiger, S., Roychoudhury, S., O'Hagan, A., Spiegelhalter, D., Neuenschwander, B.: Robust meta-analytic-predictive priors in clinical trials with historical control information. Biometrics **70**(4), 1023–1032 (2014)
