# Supplementary material for: Classifying information-sharing methods
Source: BMC Med Res Methodol. 2021 May 22;21:107. doi: 10.1186/s12874-021-01292-z (PMC8140466; doi:10.1186/s12874-021-01292-z)
Supplement: Supplementary file 3 — Additional file 3 Brief summary of each included study. Multi-page tables including a brief description of how each of the included papers shared information between direct and indirect information, on which PICOS dimension and its main characteristics. [file 12874_2021_1292_MOESM3_ESM.pdf]

Additional file 3: Summary tables of the included studies

Georgios F. Nikolaidis

March 2021

Table 1: *A brief summary of the papers included in the citation-mining review*

Reference	Summary	Ref in text
Achana et al., 2014	They extend network meta-analytic models to the multiple outcomes setting, allowing for strength to be borrowed across different outcomes. They also explain how the effect of specific interventions for particular outcomes can be predicted even if no studies assessed the intervention in question for the outcome of interest. Their approach is based on ideas originally developed by Dumouchel and Harris (1983) and shares information across interventions and outcomes simultaneously.	79
Achana et al., 2013	They extend methods that adjust for baseline-risk imbalances from the meta-analysis to the network meta-analytic framework. The models that they impose on the study-specific baselines, effectively share information across studies that enrol different populations (hence the baseline imbalances). They also describe models that can be imposed on the comparison-specific meta-regression slopes thus also sharing information across treatment comparisons.	36
Ades et al., 2010	They describe models that simultaneously analyse multiple mutually exclusive outcomes, specifically accounting for the negative, within-trial, correlations that are induced by this data structure.	80
Ades et al., 2008	This was also one of the seminal papers included in the citation-mining review. The authors discuss multi-parameter evidence synthesis and the concept of borrowing strength describing models that have previously been suggested in the literature such the confidence profile method that was proposed to adjust for bias by directly modifying the likelihood of the data, hierachical models and multi-variate approaches.	62
Ades et al., 2006	This was also one of the seminal papers included in the citation-mining review. The authors discuss the role of Bayesian methods that can accommodate borrowing of strength in cost-effectiveness analysis. They conceptually describe methods, previously described in the literature, that can simultaneously analyse multiple outcomes, share information across patient subgroups, incorporate observational evidence and be used for bias-adjustment.	25
Ades and Sutton, 2006	They describe approaches for multi-parameter evidence synthesis including the confidence profile method, cross-design synthesis, hierarchical models and functions of parameters.	8

Bujkiewicz et al., 2016	They suggest multivariate models that simultaneously analyse surrogate and final outcomes. The models they suggest can impose structure to the covariance matrix to accommodate both cases where all outcomes are related to each other and cases where some outcomes are conditionally independent.	82
Bujkiewicz et al., 2014	They expand the evidence base by adding evidence on outcomes different than the target outcomes and simultaneously synthesise them using multivariate methods; thus, borrowing strength from related outcomes. In addition, they utilise a different dataset to derive informative prior distributions for the between-studies correlations.	81
Chaimani and Salanti, 2012	They describe models that estimate and adjust for small-studies bias, thus sharing information across studies of different designs. They also suggest models that can be imposed on the comparison-specific coefficients (i.e. the comparison-specific extend of the small-study effect modification), hence sharing information across treatment comparisons.	67
Cooper et al., 2009	They propose modelling approaches for the comparison-specific effect modification coefficients (i.e. slopes) in meta-regression models.	51
Copas et al., 2018	They explore the combination of primary and secondary outcomes using multivariate Random-Effects (RE) meta-analytic models. They further show that, usually, the extent of the information gain using multivariate approaches is usually only modest.	83
da Costa et al., 2017	They describe an application of network meta-analytic models in which they assume a linear (on the modelling scale - log relative dosage) dose-response curve; thus, sharing information across treatment comparisons. They also impose a random-walk across the relative effects of different follow-ups hence sharing information across different outcomes.	43
Dakin et al., 2011	They describe models to relate outcomes that pertain to measurements taken at different parts during the day (This is here considered sharing information across different endpoints). They further model interventions within treatment classes, allowing strength to be borrowed from interventions that function through similar mechanisms.	32

Daniels and Hughes, 1997	They propose multivariate meta-analytic models that evaluate the association between surrogate markers and final outcomes. Information is shared across the two outcomes by modelling their correlation structure.	84
Del Giovane et al., 2013	They describe network meta-analytic models that relate the relative effects of different dosages of the same treatment. Specifically, they explore lumping all dosages, imposing random-walks, constraints, dose-response curves and class-effects.	39
Dias et al., 2010	They describe meta-regression type models that simultaneously analyse studies in different levels of risk-of-bias (This is considered here as studies of different design). Their model can estimate and internally adjust for biases that are due to both active vs inactive and active vs active treatment comparisons.	65
Dias et al., 2011a	They set out the basic model for Network Meta-Analysis (NMA) in which the between-trial heterogeneities are assumed to be comparison independent; Hence, information is then shared across different treatment comparisons both as part of the consistency equations of the model and as part of the common heterogeneity component.	110
Dias et al., 2011b	They describe models that explore and explain heterogeneity by accounting for specific effect modifiers. They also describe models that can be imposed on the comparison-specific effect modification coefficients to assist their identification.	54
Dias et al., 2011c	They describe models that can be imposed across the study-specific baseline parameters such as a simple random-effect across all baselines. This is considered here as sharing information amongst different populations, because the baseline imbalances may be indicative of different types of populations enrolled in different trials.	37
Ding and Fu, 2013	They describe a longitudinal model that combines information from studies that report at multiple/different follow-ups periods without the need for data reconstruction, whilst allowing prediction of relative effects pertaining to follow-ups that have not been observed.	76
Dominici et al., 1999	They describe a meta-analytic model that allows the relative treatment effects of interventions that fall under the same ‘class’ to shrink towards their class-specific mean. Hence, this assumption shares information across multiple treatment comparisons.	46

Duarte et al., 2017	Even though they seek to make a decision for a paediatric population, the authors extend the evidence base to include adult evidence and analyse the full evidence set assuming no differences across adult and paediatric patients.	14
Eddy et al., 1990	They describe the confidence profile method which adjusts for known sources of bias by directly modifying the likelihood function. This is categorised here as enabling information-sharing across studies pertaining to different designs.	72
Efthimiou et al., 2014	They describe multivariate approaches to simultaneously model multiple outcomes in the NMA.	85
Efthimiou et al., 2017	They describe models to simultaneously synthesise evidence pertaining to several study-designs. The suggested models include hierarchical models, informative prior models and design-adjusted models.	55
Efthimiou et al., 2015	They describe two multivariate approaches to simultaneously model multiple outcomes in the NMA setting. The first approach models within- and between- trial correlations separately, and the second expands the alternative model suggested by Riley et al. (2008), which only models the overall correlation, from Meta-Analysis (MA) to NMA.	86
Gamalo-Siebers et al., 2017	They describe prior-based and hierarchical methods (including power-priors) to combine paediatric and adult evidence; thus, sharing information amongst multiple populations.	34
Higgins and Whitehead, 1996	This was also one of the seminal papers included in the citation-mining review. The authors describe the standard RE NMA model and in addition, suggest a novel method for using historical information to derive an informative prior for the between-studies heterogeneity. This can be particularly helpful when evidence is sparse and heterogeneity cannot be appropriately estimated.	24
Hong et al., 2018a	They improve the alternative model suggested by Riley et al. (2008), which models a single correlation instead of within-studies and between-studies correlations, by suggesting a robust variance estimator.	88
Hong et al., 2016	They describe contrast-based and arm-based parametrisations of a framework that allows simultaneous synthesis of multiple outcomes. This framework assumes that all studies can contain all treatment arms and hence considers missing arms as missing data and imputes for them.	87

Hong et al., 2018b	Described power and commensurate prior methods to combine aggregate-level and individual-patient level evidence in NMA.	119
Hwang and DeSantis, 2018	They demonstrate that, just as in the MA setting, the use of multivariate methods has the capacity to reduce outcome reporting bias under several outcome missingness scenarios in the NMA setting as well.	89
Jackson et al., 2011	This was also one of the seminal papers included in the citation-mining review. Based on previous tutorials on multivariate methods, the authors explained multivariate meta-analysis for multiple outcomes including within- and between- studies level models and discussed potential benefits and areas of application as well as assumptions and disadvantages.	26
Jackson et al., 2013	They propose a method for multivariate RE meta-analysis that is also able to accommodate the inclusion of covariates through meta-regression.	91
Jackson et al., 2014	They propose a multivariate method to model studies that report survival outcomes at multiple/different follow-up points. Their method models the between-study covariance matrix across different time periods.	77
Jackson and Riley, 2014	They extend a refined method, previously developed by Hartung and Knapp (2001) in the univariate setting, to the multivariate setting where multiple outcomes are simultaneously modelled. This method is particularly useful when only few studies are included in the MA causing problems in the estimation of the between-studies covariance matrix.	92
Jackson et al., 2017	They describe multivariate NMA methods and further propose a method for calculating the extent of strength that is borrowed across outcomes. Their method is based on a comparison of the precision of the estimates under the univariate and the multivariate approach.	115
Jackson et al., 2018	They extend univariate NMA methods to the multivariate setting where multiple outcomes are simultaneously synthesised. Their model further allows for two types of variance components: one that is due to between-study heterogeneity and another which is due to inconsistency.	90
Kirkham et al., 2012	They show that multivariate meta-analytic methods have the capacity to reduce outcome reporting bias under several outcome missingness mechanisms.	93

Langford et al., 2018	They developed methods to meta-analyse studies reporting for different/multiple dosages of the same treatment; hence, sharing information across the relative effectiveness of different treatment comparisons. Their method utilises the Emax model that is commonly employed in pharmacology and has several advantages over other, unbounded, approaches such as linear dose-response models.	42
Liu et al., 2018	They develop a multivariate method for simultaneous synthesis of multiple outcomes where within- and between-trials correlations are accounted using copulas.	94
Lu et al., 2007	They extend NMA methods to accommodate cases where the available studies report for multiple/different fixed follow-up periods; hence, their methods borrows strength across different endpoints which is considered here as information-sharing across different outcomes.	75
Lu and Ades, 2009	They model between-trial variance structures that are compatible with consistency assumptions and allow one to incorporate prior information on correlations between treatment arms.	53
Lu et al., 2014	They suggest methods to model the treatment comparison-specific between-trials heterogeneities such as the use of triangle inequalities which stem from second order consistency.	95
Madan et al., 2014	They develop a method to simultaneously analyse multiple outcomes reported at different/several follow-ups of complex interventions. Their model shares information across outcomes and treatment comparisons simultaneously.	30
Mak et al., 2009	They use observational evidence to derive an informative prior that is used for the analysis of the available randomised trials. This is a two-step process which results in information-sharing across studies of different designs.	59
Mavridis and Salanti, 2013	They provide a thorough introduction to multivariate meta-analytic methods and a tutorial on how to simultaneously analyse multiple outcomes.	96
Mavridis et al., 2013	They describe an extension of a selection model, previously suggested by Copas (1999) that can be used in MA to account for publication bias that at is due to studies' treatment effect size and precision. This is considered here as sharing information across studies pertaining to different designs.	69

Mawdsley et al., 2016	They describe model-based NMA which simultaneously analyses trials that report for multiple dosages of a specific treatment. Their model enables information-sharing across multiple treatment comparisons using the Emax model which is commonly used in pharmacology/pharmacokinetics.	38
McCarron et al., 2010	They describe methods to combine randomised and non-randomised evidence adjusting for imbalances across study arms within studies. Two approaches are used. One that extends the model previously suggested by Prevost et al. (2000) and is essentially a three-level hierarchical model, and another that initially meta-analyses the non-randomised evidence and subsequently uses the posterior conclusions as informative priors for the analysis of the randomised evidence.	60
McCarron et al., 2011	They describe a simulation study that compares the methods presented in McCarron et al., 2010. These include multi-level models and prior-based methods to combine randomised and non-randomised evidence, that share information across different designs, accounting for imbalances across study arms.	61
Melendez-Torres et al., 2015	They discuss emergent methods for modelling complex interventions by grouping them into 'clinically meaningful units' or, in other words, according to the components of interventions that they include.	50
Mills et al., 2012	They described methods that model complex interventions by assuming additivity of the relative effects of the various components on the modelling scale. This approach shares information across treatment comparisons and also enables the evaluation of treatment combinations that have not been used in practice.	49
Moreno et al., 2011	They propose a meta-regression method that accounts for publication bias and small-study effects by regressing the treatment effect on its associated variance. The model simultaneously analyses evidence pertaining to 12 interventions, all of which fall into the same 'class' of antidepressants. Their meta-regression model also assumes exchangeability across the treatment comparison-specific meta-regression slopes. Overall, it shares information across different study designs (small/large studies) and treatment comparisons (class of antidepressants)	47

Musekiwa et al., 2016	They describe a generalised linear mixed model that can simultaneously model studies reporting at multiple pre-determined timepoints (i.e. follow-ups) accounting for within- and between-studies correlations. This model is considered to share information across several outcomes.	78
Nam et al., 2003	They suggest multivariate models that can simultaneously model and share information across multiple outcomes. Two of their models, extend the traditional univariate approach and differ in the assumptions they make at the between-studies level; the third model is a mixed model approach. They compare their approaches using a simulation experiment.	97
Nixon et al., 2007	They suggest methods to model complex interventions. These include meta-regression approaches that assume additive effects among treatment components and also a bivariate approach. They also try a class-effects model where treatments are lumped within classes. All their models share information across parameters pertaining to different treatment comparisons.	45
Owen et al., 2015	They develop a multi-level approach that models interventions within classes of treatments allowing the relative effects of each treatment to shrink toward their class-specific mean. They also impose constraints on the dosages, forcing larger dosages to exhibit larger relative effects. Their models primarily share information across parameters that pertain to different treatment comparisons.	40
Prevost et al., 2000	They suggest a hierarchical, multi-level, approach to model studies pertaining to different study designs (e.g. randomised and non-randomised studies). This includes initially modelling studies within each design and subsequently modelling design-specific hyperparameters by allowing them to shrink towards an overall design-independent hypermean. This approach also allows for separate heterogeneity components to be estimated within each design and across all designs. Their model shares information across studies of different designs.	58
Pullenayegum, 2011	They suggest the use of informative prior distributions for the between-study heterogeneneity when RE meta-analyses analyse sparse evidence. Their priors are derived based on previous meta-analyses and hence this is a meta-epidemiological approach.	107
Ren et al., 2018	They develop a method to elicit informative prior distributions that can be used for the between-trials heterogeneity in RE meta-analyses.	109

Rhodes et al., 2015	They use previous meta-analyses to obtain informative priors that can be used for the between-trials heterogeneity when the number of studies analysed with a random-effect is small and estimation of the between-studies heterogeneity becomes problematic.	108
Rietbergen, 2016	They describe the use of power-priors in many settings. In one of their applications they demonstrate how power-priors can be used to combine randomised and observational evidence by discounting the likelihood of the observational data. Their models share information across multiple study-designs.	57
Riley et al., 2007a	They describe how standard bivariate meta-analysis models can be used and compare them with the univariate approach under a set of scenarios where studies report either complete information on all outcomes or some outcomes are missing at random.	98
Riley et al., 2007b	They describe multivariate RE meta-analytic methods to simultaneously model multiple outcomes and further focus on issues that arise with the estimation of the between covariance matrix, particularly when only few studies are available and the within-study variance is large.	99
Riley et al., 2008	They describe an alternative bivariate random effect model to analyse multiple outcomes when within-trial correlations are unknown. This model does not distinguish between within-trial and between-trials correlations, and models it as a single correlation, so requires the same data as separate univariate meta-analyses. Hong et al. (2018a) showed that this model may not always appropriately estimate variance and suggested a robust variance estimator that improved on this model.	100
Roever et al., 2019	They demontrate how mixture priors can be used to combine adult and paediatric evidence where the adult evidence are part of the prior. They further show that this approach is robust to 'prior data conflict' (that is cases where direct and indirect evidence are in disagreement) and that therefore mixture priors facilitate adaptive borrowing of strength.	33
Salanti et al., 2010	They develop network meta-regression models to estimate and adjust for novelty bias in which the effectiveness of newer treatments is potentially exaggerated. This is considered here as sharing information across studies pertaining to different designs.	70

Salanti et al., 2009	They develop a network meta-regression model that estimates and adjusts for the effect modification caused by the year of publication. This is considered here a characteristic of the study-design and hence this model shares information across studies of different designs.	71
Schmitz et al., 2013	They suggest modelling approaches to combine randomised and non-randomised studies. These include a simple lumping approach where no differences are considered, using the non-randomised evidence as prior information, and analysing both sources with a three-level model that initially models studies within each design and subsequently combines the design-specific hyperparameters.	56
Soares et al., 2014	They describe modelling approaches that can be used to overcome issues relating to evidence sparsity. Amongst the suggested models there are methods that lump across different population subgroups (patients of different disease severity) and methods that impose a 'class-effect' on intervention functioning through the same mechanism.	35
Spiegelhalter and Best, 2003	They describe a modelling approach that can be used in random-effect meta-analysis to adjust for internal and external biases and therefore combine studies that may pertain to several different study-designs.	64
Tan et al., 2018	They use a bivariate meta-analytic model to obtain estimates required for decision-making that have not been reported and would not be obtainable using standard methods.	101
Thorlund et al., 2013	They conduct a simulation experiment to compare different models, originally suggested by Lu and Ades (2009), that can be imposed on the treatment comparison-specific between-trial heterogeneities. These models share information across different treatment comparisons.	52
Trinquart et al., 2012	They describe meta-regression models, similar to those suggested by Chaimani and Salanti (2012) that can be used, to estimate and adjust for reporting bias. This is assumed to be linked with the study size and therefore their models share information across studies of different designs.	68

Turner et al., 2015	They utilise meta-epidemiological data from previous meta-analyses in order to obtain 'of-the-shelf' informative priors for the between-trials heterogeneity in RE meta-analyses. These informative priors are particularly useful when there are only few studies in the meta-analysis and the estimation of the between-studies heterogeneity becomes problematic.	106
Turner et al., 2009	They suggest bias-adjustment methods which facilitate the synthesis of studies that differ in rigour (i.e. internal validity) and relevance (i.e. external validity). Their approaches allow for both additive and proportional biases on the modelling scale. These models share information across different study designs.	63
van Houwelingen et al., 2002	They describe extensions to the univariate approach (that can only model one outcome at a time). These include bivariate methods that simultaneously model two outcomes and allow information to be shared across outcomes at the within- and the between-studies level.	103
van Houwelingen et al., 1993	The authors set the initial ideas around the use of multivariate meta-analysis to simultaneously model multiple outcomes allowing strength to be borrowed across outcomes through their correlation structure.	102
Warren et al., 2014	They describe how hierarchical methods can be used to model multiple dosages of the same interventions and multiple treatments that fall under the same 'class' (i.e. mechanism of action). Furthermore, they show how dosage constraints can be imposed assuming that larger dosages exhibit larger relative effects.	44
Wei and Higgins, 2013a	They suggest an approach that can be used for multivariate models to approximate within-study covariances when their estimation is problematic because the within-trial correlations are either unknown or cannot be estimated using Individual-patient data (IPD).	104
Wei and Higgins, 2013b	They set out to extend bivariate meta-analytic methods to cases where more than two outcomes are simultaneously modelled. They further suggest alternatives to the Wishart prior for the variance-covariance matrix and explore simplifying assumptions that can be imposed on the variances and the correlations when their number increases due to additional outcomes included in the analysis.	105

Welton et al., 2009b	They suggest network meta-regression approaches that can be used to model complex interventions with multiple treatment components. On top of simple additive -on the modelling scale- relative effects, they also show how interaction effects (i.e. synergistic or antagonistic) can be incorporated in the model.	48
Welton et al., 2009a	They suggest hierarchical models that can be used to simultaneously model studies in high and low risk of bias using a bias-adjustment approach; hence, their models share information across multiple study-designs. They further show how external evidence can be used to derive informative priors for the bias component.	66
Welton et al., 2008	They suggest models that simultaneously synthesize two structurally related time-to-event outcomes. They use constraints to reflect that one endpoint needs to be reached before the other, and they also model their between-studies covariance using multivariate methods.	74
Welton et al., 2010	They develop a multi-parameter evidence synthesis framework to model multiple time-to event outcomes. They reflect structural relationships among outcomes by forcing their relative treatment effects to differ by a fixed component term. They also reflect the between-study correlation structure amongst outcomes using multivariate methods.	31
Wolpert and Kerrie, 2004	They suggest models, similar to those developed by Eddy et al. (1990), to model multiple studies pertaining to several designs by directly modelling sources of bias using adjusted likelihoods.	73
Wu et al., 2018	They describe methods for model-based meta-analysis of biologic products using a linear dose-response relationship where the dosage is proportional to the relative effect -on the modelling scale- and also using the commonly employed in the pharmacokinetics field non-linear Emax model. Their models share information across treatment comparisons (i.e. the relative effects of different dosages of the same treatment).	41

Table 2: *A summary table of the main characteristics of the included studies.*

Reference	Challenge(s)	Cores	PICOS	Method(s)	Parameter(s)	MA/NMA	Ref. in text
Achana et al., 2014	Ch.11	Multivariate	Outcome	BW	RTE	NMA	79
Achana et al., 2013	Ch.3	Functional, Exchangeability, Priors	Treatment comparisons, Populations	RE, L, MixP	RTE, baselines, meta-regression slopes	NMA	36
Ades et al., 2010	Ch.11	Multivariate	Outcomes	W	RTE	NMA	80
Ades et al., 2008	Ch.7, Ch. 11	Functional, Exchangeability, Multivariate	Populations, Treatment comparisons, Outcomes, Designs	L, MLM, BW	RTE	MA/NMA	62
Ades et al., 2006	Ch.7, Ch. 11	Functional, Exchangeability, Multivariate	Populations, Treatment comparisons, Outcomes, Designs	L, MLM, BW	RTE	MA/NMA	25
Ades and Sutton, 2006	Ch.7, Ch. 11	Functional, Exchangeability, Multivariate	Populations, Treatment comparisons, Outcomes, Designs	L, MLM, BW	RTE	MA/NMA	8

Bujkiewicz et al., 2016	Ch. 11	Multivariate	Outcomes	BW	RTE	MA	82
Bujkiewicz et al., 2014	Ch. 11	Multivariate	Outcomes	BW	RTE	MA	81
Chaimani and Salanti, 2012	Ch.8	Functional, Exchangeability	Treatment comparisons, Designs	RE, L	RTE	NMA	67
Cooper et al., 2009	Ch. 14	Functional, Exchangeability	Treatment comparisons	Lumping, RE	meta-regression slopes	NMA	51
Copas et al., 2018	Ch. 11	Multivariate	Outcomes	BW	RTE	MA	83
da Costa et al., 2017	Ch.4, Ch.10	Functional, Exchangeability	Outcomes, Treatment comparisons	L, RW	RTE	NMA	43
Dakin et al., 2011	Ch.5	Functional, Exchangeability	Outcomes, Treatment comparisons	L, MLM	RTE	NMA	32
Daniels and Hughes, 1997	Ch.11	Multivariate	Outcomes	BW	RTE	MA	84
Del Giovane et al., 2013	Ch.4	Functional, Exchangeability	Treatment comparisons	C, RE, RW	RTE	NMA	39
Dias et al., 2010	Ch.8	Functional	Designs	L	RTE	NMA	65

Dias et al., 2011b	Ch.14	Functional	Treatment compar-isons	L	meta-regression slopes	NMA	54
Dias et al., 2011c	Ch.3	Exchangeability	Populations	RE	baselines	NMA	37
Ding and Fu, 2013	Ch.10	Functional	Outcomes	NL	RTE	NMA	76
Dominici et al., 1999	Ch.5	Exchangeability	Treatment compar-isons, Designs	MLM	RTE	NMA	46
Duarte et al., 2017	Ch.1	Functional	Populations	Lumping	RTE	NMA	14
Eddy et al., 1990	Ch.8	Functional	Designs	L	RTE	MA	72
Efthimiou et al., 2014	Ch.11, Ch.13	Multivariate	Outcomes	BW	RTE	NMA	85
Efthimiou et al., 2017	Ch.7, Ch.8	Exchangeability, Prior	Designs	MLM, SIP	RTE	NMA	55
Efthimiou et al., 2015	Ch.11	Multivariate	Outcomes	BW, S	RTE	NMA	86
Gamalo-Siebers et al., 2017	Ch.1	Exchangeability, Prior	Populations	MLM, SIP, PP,	RTE	MA	34
Higgins and Whitehead, 1996	Ch.12	Prior	FOO	SIP	Heterogeneity	MA	24

Hong et al., 2018a	Ch.11	Multivariate	Outcomes	S	RTE	MA	88
Hong et al., 2016	Ch.11	Multivariate	Outcomes	B	RTE	NMA	87
Hwang and DeSantis, 2018	Ch.11	Multivariate	Outcomes	BW	RTE	NMA	89
Jackson et al., 2011	Ch.11	Multivariate	Outcomes	BW	RTE	MA	26
Jackson et al., 2013	Ch.11	Multivariate	Outcomes	BW	RTE	MA	91
Jackson et al., 2014	Ch.10	Multivariate	Outcomes	BW	RTE	MA	77
Jackson and Riley, 2014	Ch.11	Multivariate	Outcomes	BW	RTE	MA	92
Jackson et al., 2017	Ch.11	Multivariate	Outcomes	BW	RTE	NMA	115
Jackson et al., 2018	Ch.11	Multivariate	Outcomes	BW	RTE	NMA	90
Kirkham et al., 2012	Ch.11	Multivariate	Outcomes	BW	RTE	MA	93
Langford et al., 2018	Ch.4	Functional	Treatment comparisons	NL	RTE	MA	42
Liu et al., 2018	Ch.11	Multivariate	Outcomes	BW	RTE	NMA	94
Lu et al., 2007	Ch.10	Functional, Exchangeability	Outcomes	NL, RW	RTE	NMA	75
Lu and Ades, 2009	Ch.14	Functional, Exchangeability	Treatment comparisons	C, RE	Heterogeneity	NMA	53

Lu et al., 2014	Ch.11	Multivariate	Outcomes	BW	RTE	MA	95
Madan et al., 2014	Ch.6, Ch.11	Functional, Multivariate	Treatment comparisions, Outcomes	L, MV	RTE	NMA	30
Mak et al., 2009	Ch.7	Priors	Designs	SIP	RTE	MA	59
Mavridis and Salanti, 2013	Ch.11	Multivariate	Outcomes	BW	RTE	MA	96
Mavridis et al., 2013	Ch.8	Functional	Designs	L	RTE	NMA	69
Mawdsley et al., 2016	Ch.4	Functional	Treatment comparisons	NL	RTE	NMA	38
McCarron et al., 2010	Ch.7	Exchangeability, Priors	Designs	MLM, SIP	RTE	NMA	60
McCarron et al., 2011	Ch.7	Exchangeability, Priors	Designs	MLM, SIP	RTE	NMA	61
Melendez-Torres et al., 2015	Ch.6	Functional	Treatment comparisons	L	RTE	NMA	50
Mills et al., 2012	Ch.6	Functional	Treatment comparisons	L	RTE	NMA	49

Moreno et al., 2011	Ch.5, Ch.8, Ch.12	Functional, Exchangeability, Priors	Treatment comparisons	L, RE, SIP	RTE, meta-regression slopes	NMA	47
Musekiwa et al., 2016	Ch.10	Multivariate	Outcomes	BW	RTE	MA	78
Nam et al., 2003	Ch.11	Multivariate	Outcomes	BW	RTE	MA	97
Nixon et al., 2007	Ch.5, Ch.6	Functional, Exchangeability, Multivariate	Treatment comparisons	L, MLM, B	RTE	NMA	45
Owen et al., 2015	Ch.4, Ch.5	Functional, Exchangeability	Treatment comparisons	C, MLM	RTE	NMA	40
Prevost et al., 2000	Ch.7	Exchangeability	Designs	MLM	RTE	MA	58
Pullenayegum, 2011	Ch.12	Prior	Meta-epi	SIP	Heterogeneity	MA	107
Ren et al., 2018	Ch.13	Prior	Elicitation	SIP	Heterogeneity	NMA	109
Rhodes et al., 2015	Ch.12	Prior	Meta-epi	SIP	Heterogeneity	MA	108
Rietbergen, 2016	Ch.7	Prior	Designs	PP	RTE	NMA	57
Riley et al., 2007a	Ch.11	Multivariate	Outcomes	BW	RTE	MA	98
Riley et al., 2007b	Ch.11	Multivariate	Outcomes	BW	RTE	MA	99

Riley et al., 2008	Ch.11	Multivariate	Outcomes	S	RTE	MA	100
Roever et al., 2019	Ch.1	Prior	Populations	MixP	RTE	MA	33
Salanti et al., 2010	Ch.8	Functional	Designs	L	RTE	NMA	70
Salanti et al., 2009	Ch.8	Functional	Designs	L	RTE	NMA	71
Schmitz et al., 2013	Ch.7	Exchangeability, Priors	Designs	MLM, SIP	RTE	NMA	56
Soares et al., 2014	Ch.2, Ch.5	Functional, Exchangeability	Populations, Treatment comparisons	L, MLM	RTE	NMA	35
Spiegelhalter and Best, 2003	Ch.8	Functional	Designs	L	RTE	NMA	64
Tan et al., 2018	Ch.11	Multivariate	Outcomes	BW	RTE	MA	101
Thorlund et al., 2013	Ch.14	Functional, Exchangeability	Treatment comparisons	C, RE	Heterogeneity	NMA	52
Trinquart et al., 2012	Ch.8	Functional	Designs	L	RTE	NMA	68
Turner et al., 2015	Ch.12	Prior	Meta-epi	SIP	Heterogeneity	MA	106
Turner et al., 2009	Ch.8	Functional	Designs	L	RTE	MA	63

van Houwelingen et al., 2002	Ch.11	Multivariate	Outcomes	BW	RTE	MA	103
van Houwelingen et al., 1993	Ch.11	Multivariate	Outcomes	BW	RTE	MA	102
Warren et al., 2014	Ch.4, Ch.5	Functional, Exchangeability	Treatment comparisons	L, MLM	RTE	NMA	44
Wei and Higgins, 2013a	Ch.11	Multivariate	Outcomes	BW	RTE	MA	104
Wei and Higgins, 2013b	Ch.11	Multivariate	Outcomes	BW	RTE	MA	105
Welton et al., 2009b	Ch.6	Functional	Treatment comparisons	L	RTE	NMA	48
Welton et al., 2009a	Ch.8, Ch.12	Functional, Prior	Designs, Meta-epi	L, SIP	RTE	NMA	66
Welton et al., 2008	Ch.9, Ch.11	Functional, Multivariate	Outcomes	C, BW	RTE	NMA	74
Welton et al., 2010	Ch.9, Ch.11	Functional, Multivariate	Outcomes	L, BW	RTE	NMA	31
Wolpert and Kerrie, 2004	Ch.8	Functional	Designs	L	RTE	MA	73
Wu et al., 2018	Ch.4	Functional	Treatment comparisons	NL	RTE	MA	41

Note:
Challenge 1: Synthesis of evidence on adults and children
Challenge 2: Synthesis of evidence pertaining to different population subgroups
Challenge 3: Synthesis of evidence on populations pertaining to different baseline risk
Challenge 4: Synthesis of evidence on multiple dosage of the same treatment
Challenge 5: Synthesis of evidence on treatments pertaining to the same class
Challenge 6: Synthesis of evidence relating to complex interventions
Challenge 7: Synthesis of randomised and non-randomised evidence
Challenge 8: Synthesis of evidence accounting for internal and/or external bias
Challenge 9: Synthesis of evidence on multiple structurally related outcomes
Challenge 10: Synthesis of evidence relating to different follow-up periods
Challenge 11: Synthesis of multiple correlated outcomes
Challenge 12: Borrowing strength from meta-epidemiological evidence
Challenge 13: Incorporating evidence from expert elicitation
Challenge 14: Synthesis of comparison-specific non-relative effect parameters such as between-studies heterogeneities and meta-regression slopes

C: Constraint, L: Linear relationship (e.g. meta-regression), N-L: Non-linear, RE: Random-Effect, RW: Random-Walk, MLM: Multi-level model, SIP: Standard Informative Prior, MixP: Mixture prior, PP: Power-prior, B: Only between-studies correlation modelled, W: Only within-study correlation modelled, B&W: Both within-study and between-studies correlations modelled separately, S: Within-study and between-studies correlations modelled simultaneously as one parameter.

Bibliography

Achana, F. A., Cooper, N. J., Dias, S., Lu, G., Rice, S. J., Kendrick, D., and Sutton, A. J. Extending methods for investigating the relationship between treatment effect and baseline risk from pairwise meta-analysis to network meta-analysis. *Stat Med*, 32(5): 752–71, 2013. ISSN 0277-6715.

Achana, F. A., Cooper, N. J., Bujkiewicz, S., Hubbard, S. J., Kendrick, D., Jones, D. R., and Sutton, A. J. Network meta-analysis of multiple outcome measures accounting for borrowing of information across outcomes. *BMC Med Res Methodol*, 14:92, 2014. ISSN 1471-2288.

Ades, A. E. and Sutton, A. J. Multiparameter evidence synthesis in epidemiology and medical decision-making: current approaches. *Journal of the Royal Statistical Society: Series A (Statistics in Society)*, 169(1):5–35, 2006. ISSN 1467-985X.

Ades, A. E., Sculpher, M., Sutton, A., Abrams, K., Cooper, N., Welton, N., and Lu, G. Bayesian methods for evidence synthesis in cost-effectiveness analysis. *PharmacoEconomics*, 24:1–19, 2006. ISSN 1170-7690.

Ades, A. E., Welton, N. J., Caldwell, D., Price, M., Goubar, A., and Lu, G. Multiparameter evidence synthesis in epidemiology and medical decision-making. *Journal of Health Services Research & Policy*, 13(3):12–22, October 2008. ISSN 1355-8196. doi: 10.1258/jhsrp.2008.008020.

Ades, A. E., Mavranezouli, I., Dias, S., Welton, N. J., Whittington, C., and Kendall, T. Network meta-analysis with competing risk outcomes. *Value Health*, 13(8):976–83, 2010. ISSN 1098-3015.

Bujkiewicz, S., Thompson, J. R., Sutton, A. J., Cooper, N. J., Harrison, M. J., Symmons, D. P. M., and Abrams, K. R. Use of bayesian multivariate meta-analysis to estimate the haq for mapping onto the eq-5d questionnaire in rheumatoid arthritis. *Value in Health*, 2014.

Bujkiewicz, S., Thompson, J. R., Riley, R. D., and Abrams, K. R. Bayesian meta-analytical methods to incorporate multiple surrogate endpoints in drug development process. *Statistics In Medicine*, 35(7, SI):1063–1089, March 2016. ISSN 0277-6715.

Chaimani, A. and Salanti, G. Using network meta-analysis to evaluate the existence of small-study effects in a network of interventions. *Research Synthesis Methods*, 3(2): 161–176, 2012. ISSN 1759-2887.

Cooper, N. J., Sutton, A. J., Morris, D., Ades, A. E., and Welton, N. J. Addressing between-study heterogeneity and inconsistency in mixed treatment comparisons: Application to stroke prevention treatments in individuals with non-rheumatic atrial fibrillation. *Statistics in Medicine*, 28(14):1861–1881, 2009. ISSN 1097-0258.

Copas, J. What works?: selectivity models and meta-analysis. *Journal of the Royal Statistical Society: Series A (Statistics in Society)*, 162(1):95–109, 1999.

Copas, J. B., Jackson, D., White, I. R., and Riley, R. D. The role of secondary outcomes in multivariate meta-analysis. *Journal of the Royal Statistical Society Series C - Applied Statistics*, 67(5):1177–1205, November 2018. ISSN 0035-9254.

da Costa, B. R., Reichenbach, S., Keller, N., Nartey, L., Wandel, S., Juni, P., and Trelle, S. Effectiveness of non-steroidal anti-inflammatory drugs for the treatment of pain in knee and hip osteoarthritis: a network meta-analysis. *Lancet*, 390(10090):E21–E33, July 2017. ISSN 0140-6736.

Dakin, H. A., Welton, N. J., Ades, A. E., Collins, S., Orme, M., and Kelly, S. Mixed treatment comparison of repeated measurements of a continuous endpoint: an example using topical treatments for primary open-angle glaucoma and ocular hypertension. *Stat Med*, 30(20):2511–35, 2011. ISSN 0277-6715.

Daniels, M. J. and Hughes, M. D. Meta-analysis for the evaluation of potential surrogate markers. *Stat Med*, 16(17):1965–82, 1997. ISSN 0277-6715.

Del Giovane, C., Vacchi, L., Mavridis, D., Filippini, G., and Salanti, G. Network meta-analysis models to account for variability in treatment definitions: application to dose effects. *Stat Med*, 32(1):25–39, 2013. ISSN 0277-6715.

Dias, S., Welton, N. J., Marinho, V. C. C., Salanti, G., Higgins, J. P. T., and Ades, A. E. Estimation and adjustment of bias in randomized evidence by using mixed treatment comparison meta-analysis. *Journal of the Royal Statistical Society: Series A (Statistics in Society)*, 173(3):613–629, 2010. ISSN 1467-985X.

Dias, S., Welton, N., Sutton, A., and Ades, A. *NICE DSU Technical Support Document 2: A Generalised Linear Modelling Framework for Pairwise and Network Meta-Analysis of Randomised Controlled Trials*. Number TSD2 in Technical Support Document in Evidence Synthesis. National Institute for Health and Clinical Excellence, 8 2011a.

Dias, S., Sutton, A., Welton, N., and Ades, A. *NICE DSU Technical Support Document 3: Heterogeneity: Subgroups, Meta-Regression, Bias and Bias-Adjustment*. National Institute for Health and Clinical Excellence, 9 2011b.

Dias, S., Welton, N., Sutton, A., and Ades, A. *NICE DSU Technical Support Document 5: Evidence Synthesis in the Baseline Natural History Model*. Number TSD5 in NICE DSU Technical Support Document in Evidence Synthesis. National Institute for Health and Clinical Excellence, 8 2011c.

Ding, Y. and Fu, H. Bayesian indirect and mixed treatment comparisons across longitudinal time points. *Stat Med*, 32(15):2613–28, 2013. ISSN 0277-6715.

Dominici, F., Parmigiani, G., Wolpert, R. L., and Hasselblad, V. Meta-analysis of migraine headache treatments: Combining information from heterogeneous designs. *Journal of the American Statistical Association*, 94(445):16–28, 1999.

Duarte, A., Mebrahtu, T., and Goncalves, P. Adalimumab, etanercept and ustekinumab for treating plaque psoriasis in children and young people: systematic review and economic evaluation. *Health Technology Assessment*, 2017.

Dumouchel, W. H. and Harris, J. E. Bayes methods for combining the results of cancer studies in humans and other species. *Journal of the American Statistical Association*, 78 (382):293–308, 1983.

Eddy, D. M., Hasselblad, V., and Shachter, R. An introduction to a bayesian method for meta-analysis: The confidence profile method. *Med Decis Making*, 10(1):15–23, 1990. ISSN 0272-989X.

Efthimiou, O., Mavridis, D., Cipriani, A., Leucht, S., Bagos, P., and Salanti, G. An approach for modelling multiple correlated outcomes in a network of interventions using odds ratios. *Statistics in Medicine*, 33(13):2275–2287, 2014. ISSN 1097-0258.

Efthimiou, O., Mavridis, D., Riley, R. D., Cipriani, A., and Salanti, G. Joint synthesis of multiple correlated outcomes in networks of interventions. *BIOSTATISTICS*, 16(1): 84–97, January 2015. ISSN 1465-4644.

Efthimiou, O., Mavridis, D., Debray, T. P. A., Samara, M., Belger, M., Siontis, G. C. M., Leucht, S., Salanti, G., and on behalf of GetReal Work, P. Combining randomized and non-randomized evidence in network meta-analysis. *Statistics in Medicine*, 36(8): 1210–1226, 2017. ISSN 1097-0258.

Gamalo-Siebers, M., Savic, J., Basu, C., Zhao, X., Gopalakrishnan, M., Gao, A., Song, G., Baygani, S., Thompson, L., Xia, H. A., Price, K., Tiwari, R., and Carlin, B. P. Statistical modeling for Bayesian extrapolation of adult clinical trial information in pediatric drug evaluation. *Pharmaceutical Statistics*, 16(4):232–249, August 2017. ISSN 1539-1604.

Hartung, J. and Knapp, G. A refined method for the meta-analysis of controlled clinical trials with binary outcome. *Statistics in Medicine*, 20(24):3875–3889, 2001.

Higgins, J. P. T. and Whitehead, A. Borrowing strength from external trials in a meta-analysis. *Statistics in Medicine*, 15(24):2733–2749, 1996. ISSN 1097-0258.

Hong, C., Riley, R. D., and Chen, Y. An improved method for bivariate meta-analysis when within-study correlations are unknown. *Research Synthesis Methods*, 9(1):73–88, March 2018a. ISSN 1759-2879.

Hong, H., Chu, H., Zhang, J., and Carlin, B. P. A bayesian missing data framework for generalized multiple outcome mixed treatment comparisons. *Research Synthesis Methods*, 7(1):6–22, 2016. ISSN 1759-2887.

Hong, H., Fu, H., and Carlin, B. P. Power and commensurate priors for synthesizing aggregate and individual patient level data in network meta-analysis. *Journal of the Royal Statistical Society: Series C (Applied Statistics)*, 2018b.

Hwang, H. and DeSantis, S. M. Multivariate network meta-analysis to mitigate the effects of outcome reporting bias. *Statistics In Medicine*, 37:3254–3266, September 2018. ISSN 0277-6715.

Jackson, D., Riley, R., and White, I. R. Multivariate meta-analysis: Potential and promise. *Statistics in Medicine*, 30(20):2481–2498, 2011. ISSN 1097-0258.

Jackson, D., White, I. R., and Riley, R. D. A matrix-based method of moments for fitting the multivariate random effects model for meta-analysis and meta-regression. *BIOMETRICAL JOURNAL*, 55(2):231–245, March 2013. ISSN 0323-3847.

Jackson, D., Rollins, K., and Coughlin, P. A multivariate model for the meta-analysis of study level survival data at multiple times. *Research Synthesis Methods*, 5(3):264–272, September 2014. ISSN 1759-2879.

Jackson, D., White, I. R., Price, M., Copas, J., and Riley, R. D. Borrowing of strength and study weights in multivariate and network meta-analysis. *Statistical Methods in Medical Research*, 26(6):2853–2868, December 2017. ISSN 0962-2802.

Jackson, D., Bujkiewicz, S., Law, M., Riley, R. D., and White, I. R. A matrix-based method of moments for fitting multivariate network meta-analysis models with multiple outcomes and random inconsistency effects. *Biometrics*, 74(2):548–556, June 2018. ISSN 0006-341X.

Jackson, D. and Riley, R. D. A refined method for multivariate meta-analysis and meta-regression. *Statistics In Medicine*, 33(4):541–554, February 2014. ISSN 0277-6715.

Kirkham, J. J., Riley, R. D., and Williamson, P. R. A multivariate meta-analysis approach for reducing the impact of outcome reporting bias in systematic reviews. *Statistics In Medicine*, 31(20):2179–2195, September 2012. ISSN 0277-6715.

Langford, O., Aronson, J. K., van Valkenhoef, G., and Stevens, R. J. Methods for meta-analysis of pharmacodynamic dose-response data with application to multi-arm studies of alogliptin. *Statistical Methods in Medical Research*, 27(2):564–578, February 2018. ISSN 0962-2802.

Liu, Y., DeSantis, S. M., and Chen, Y. Bayesian mixed treatment comparisons meta-analysis for correlated outcomes subject to reporting bias. *Journal of the Royal Statistical Society Series C - Applied Statistics*, 67(1):127–144, January 2018. ISSN 0035-9254.

Lu, G. and Ades, A. Modeling between-trial variance structure in mixed treatment comparisons. *Biostatistics*, 10(4):792–805, 2009. ISSN 1465-4644.

Lu, G., Ades, A. E., Sutton, A. J., Cooper, N. J., Briggs, A. H., and Caldwell, D. M. Meta-analysis of mixed treatment comparisons at multiple follow-up times. *Stat Med*, 26 (20):3681–99, 2007. ISSN 0277-6715.

Lu, G., Kounali, D., and Ades, A. E. Simultaneous Multioutcome Synthesis and Mapping of Treatment Effects to a Common Scale. *VALUE IN HEALTH*, 17(2):280–287, March 2014. ISSN 1098-3015.

Madan, J., Chen, Y.-F., Aveyard, P., Wang, D., Yahaya, I., Munafo, M., Bauld, L., and Welton, N. Synthesis of evidence on heterogeneous interventions with multiple outcomes recorded over multiple follow-up times reported inconsistently: a smoking cessation case-study. *Journal of the Royal Statistical Society: Series A (Statistics in Society)*, 177 (1):295–314, 2014. ISSN 1467-985X.

Mak, A., Cheung, M. W. L., Ho, R. C.-M., Cheak, A. A.-C., and Lau, C. S. Bisphosphonates and atrial fibrillation: Bayesian meta-analyses of randomized controlled trials and observational studies. *BMC Musculoskeletal Disorders*, 10, September 2009. ISSN 1471-2474.

Mavridis, D. and Salanti, G. A practical introduction to multivariate meta-analysis. *Stat Methods Med Res*, 22(2):133–58, 2013. ISSN 0962-2802.

Mavridis, D., Sutton, A., Cipriani, A., and Salanti, G. A fully bayesian application of the copas selection model for publication bias extended to network meta-analysis. *Stat Med*, 32(1):51–66, 2013. ISSN 0277-6715.

Mawdsley, D., Bennetts, M., Dias, S., Boucher, M., and Welton, N. J. Model-based network meta-analysis: A framework for evidence synthesis of clinical trial data. *CPT Pharmacometrics Syst Pharmacol*, 5(8):393–401, 2016. ISSN 2163-8306.

McCarron, C. E., Pullenayegum, E. M., Thabane, L., Goeree, R., and Tarride, J.-E. The importance of adjusting for potential confounders in Bayesian hierarchical models synthesising evidence from randomised and non-randomised studies: an application comparing treatments for abdominal aortic aneurysms. *BMC Medical Research Methodology*, 10, July 2010. ISSN 1471-2288.

McCarron, C. E., Pullenayegum, E. M., Thabane, L., Goeree, R., and Tarride, J.-E. Bayesian Hierarchical Models Combining Different Study Types and Adjusting for Covariate Imbalances: A Simulation Study to Assess Model Performance. *PLOS ONE*, 6(10), October 2011. ISSN 1932-6203.

Melendez-Torres, G. J., Bonell, C., and Thomas, J. Emergent approaches to the meta-analysis of multiple heterogeneous complex interventions. *BMC Medical Research Methodology*, 15, June 2015. ISSN 1471-2288.

Mills, E. J., Thorlund, K., and Ioannidis, J. P. A. Calculating additive treatment effects from multiple randomized trials provides useful estimates of combination therapies. *Journal of Clinical Epidemiology*, 65(12):1282–1288, December 2012. ISSN 0895-4356.

Moreno, S. G., Sutton, A. J., Ades, A. E., Cooper, N. J., and Abrams, K. R. Adjusting for publication biases across similar interventions performed well when compared with gold standard data. *Journal of Clinical Epidemiology*, 64(11):1230–1241, November 2011. ISSN 0895-4356.

Musekiwa, A., Manda, S. O. M., Mwambi, H. G., and Chen, D.-G. Meta-Analysis of Effect Sizes Reported at Multiple Time Points Using General Linear Mixed Model. *PLOS ONE*, 11(10), October 2016. ISSN 1932-6203.

Nam, I., Mengersen, K., and Garthwaite, P. Multivariate meta-analysis. *Statistics In Medicine*, 22(14):2309–2333, July 2003. ISSN 0277-6715.

Nixon, R. M., Bansback, N., and Brennan, A. Using mixed treatment comparisons and meta-regression to perform indirect comparisons to estimate the efficacy of biologic treatments in rheumatoid arthritis. *Stat Med*, 26(6):1237–54, 2007. ISSN 0277-6715.

Owen, R. K., Tincello, D. G., and Keith, R. A. Network meta-analysis: development of a three-level hierarchical modeling approach incorporating dose-related constraints. *Value Health*, 18(1):116–26, 2015. ISSN 1098-3015.

Prevost, T., Abrams, K., and Jones, D. Hierarchical models in generalized synthesis of evidence: an example based on studies of breast cancer screening. *Statistics In Medicine*, 19(24):3359–3376, December 2000. ISSN 0277-6715.

Pullenayegum, E. M. An informed reference prior for between-study heterogeneity in meta-analyses of binary outcomes. *Statistics In Medicine*, 30(26):3082–3094, November 2011. ISSN 0277-6715.

Ren, S., Oakley, J. E., and Stevens, J. W. Incorporating Genuine Prior Information about Between-Study Heterogeneity in Random Effects Pairwise and Network Meta-analyses. *Medical Decision Making*, 38(4):531–542, May 2018. ISSN 0272-989X.

Rhodes, K. M., Turner, R. M., and Higgins, J. P. T. Predictive distributions were developed for the extent of heterogeneity in meta-analyses of continuous outcome data. *Journal of Clinical Epidemiology*, 68(1):52–60, January 2015. ISSN 0895-4356.

Rietbergen, C. *Quantitative Evidence Synthesis with Power Priors*. PhD thesis, University of Utrecht, 2016.

Riley, R. D., Abrams, K. R., Lambert, P. C., Sutton, A. J., and Thompson, J. R. An evaluation of bivariate random-effects meta-analysis for the joint synthesis of two correlated outcomes. *Statistics In Medicine*, 26(1):78–97, January 2007a. ISSN 0277-6715.

Riley, R. D., Thompson, J. R., and Abrams, K. R. An alternative model for bivariate random-effects meta-analysis when the within-study correlations are unknown. *Biostatistics*, 9(1): 172–86, 2008. ISSN 1465-4644.

Riley, R. D., Abrams, K. R., Sutton, A. J., Lambert, P. C., and Thompson, J. R. Bivariate random-effects meta-analysis and the estimation of between-study correlation. *BMC Medical Research Methodology*, 7, January 2007b. ISSN 1471-2288.

Roever, C., Wandel, S., and Friede, T. Model averaging for robust extrapolation in evidence synthesis. *Statistics In Medicine*, 38(4, SI):674–694, February 2019. ISSN 0277-6715.

Salanti, G., Marinho, V., and Higgins, J. P. A case study of multiple-treatments meta-analysis demonstrates that covariates should be considered. *J Clin Epidemiol*, 62(8): 857–64, 2009. ISSN 0895-4356.

Salanti, G., Dias, S., Welton, N. J., Ades, A. E., Golfinopoulos, V., Kyrgiou, M., Mauri, D., and Ioannidis, J. P. Evaluating novel agent effects in multiple-treatments meta-regression. *Stat Med*, 29(23):2369–83, 2010. ISSN 0277-6715.

Schmitz, S., Adams, R., and Walsh, C. Incorporating data from various trial designs into a mixed treatment comparison model. *Statistics in Medicine*, 32(17):2935–2949, 2013. ISSN 1097-0258.

Soares, M. O., Dumville, J. C., Ades, A. E., and Welton, N. J. Treatment comparisons for decision making: facing the problems of sparse and few data. *Journal of the Royal Statistical Society: Series A (Statistics in Society)*, 177(1):259–279, 2014. ISSN 1467-985X.

Spiegelhalter, D. J. and Best, N. G. Bayesian approaches to multiple sources of evidence and uncertainty in complex cost-effectiveness modelling. *Stat Med*, 22(23):3687–709, 2003. ISSN 0277-6715.

Tan, S. H., Abrams, K. R., and Bujkiewicz, S. Bayesian Multiparameter Evidence Synthesis to Inform Decision Making: A Case Study in Metastatic Hormone-Refractory Prostate Cancer. *Medical Decision Making*, 38(7):834–848, October 2018. ISSN 0272-989X.

Thorlund, K., Thabane, L., and Mills, E. J. Modelling heterogeneity variances in multiple treatment comparison meta-analysis. are informative priors the better solution? *BMC Medical Research Methodology*, 13:2–2, 2013. ISSN 1471-2288.

Trinquart, L., Chatellier, G., and Ravaud, P. Adjustment for reporting bias in network meta-analysis of antidepressant trials. *BMC Medical Research Methodology*, 12:150–150, 2012. ISSN 1471-2288.

Turner, R. M., Spiegelhalter, D. J., Smith, G. C. S., and Thompson, S. G. Bias modelling in evidence synthesis. *Journal of the Royal Statistical Society: Series A (Statistics in Society)*, 172(1):21–47, 2009. ISSN 1467-985X.

Turner, R. M., Jackson, D., Wei, Y., Thompson, S. G., and Higgins, J. P. T. Predictive distributions for between-study heterogeneity and simple methods for their application

in Bayesian meta-analysis. *Statistics In Medicine*, 34(6):984–998, March 2015. ISSN 0277-6715.

van Houwelingen, H. C., Arends, L. R., and Stijnen, T. Advanced methods in meta-analysis: multivariate approach and meta-regression. *Statistics in medicine*, 21:589–624, Feb 2002. ISSN 0277-6715.

van Houwelingen, H., Zwinderman, K., and Stijnen, T. A bivariate approach to meta-analysis. *Statistics In Medicine*, 12:2273–2284, December 1993. ISSN 0277-6715.

Warren, F. C., Abrams, K. R., and Sutton, A. J. Hierarchical network meta-analysis models to address sparsity of events and differing treatment classifications with regard to adverse outcomes. *Statistics In Medicine*, 33(14):2449–2466, 2014. ISSN 1097-0258.

Wei, Y. and Higgins, J. P. T. Estimating within-study covariances in multivariate meta-analysis with multiple outcomes. *Statistics In Medicine*, 32(7):1191–1205, March 2013a. ISSN 0277-6715.

Wei, Y. and Higgins, J. P. Bayesian multivariate meta-analysis with multiple outcomes. *Statistics in Medicine*, 32(17):2911–2934, 2013b. ISSN 1097-0258.

Welton, N. J., Cooper, N. J., Ades, A. E., Lu, G., and Sutton, A. J. Mixed treatment comparison with multiple outcomes reported inconsistently across trials: evaluation of antivirals for treatment of influenza a and b. *Stat Med*, 27(27):5620–39, 2008. ISSN 0277-6715.

Welton, N. J., Ades, A. E., Carlin, J. B., Altman, D. G., and Sterne, J. A. C. Models for Potentially Biased Evidence in Meta-Analysis Using Empirically Based Priors. *Journal of the Royal Statistical Society. Series A (Statistics in Society)*, 172(1):119–136, 2009a. ISSN 0964-1998.

Welton, N. J., Caldwell, D. M., Adamopoulos, E., and Vedhara, K. Mixed treatment comparison meta-analysis of complex interventions: psychological interventions in coronary heart disease. *Am J Epidemiol*, 169(9):1158–65, 2009b. ISSN 0002-9262.

Welton, N. J., Willis, S. R., and Ades, A. E. Synthesis of survival and disease progression outcomes for health technology assessment of cancer therapies. *Research Synthesis Methods*, 1(3-4):239–257, 2010. ISSN 1759-2887.

Wolpert, R. L. and Kerrie, L. M. Adjusted likelihoods for synthesizing empirical evidence from studies that differ in quality and design: Effects of environmental tobacco smoke. *Statistical Science*, 19(3):450–471, 2004. ISSN 08834237.

Wu, J., Banerjee, A., Jin, B., Menon, S. M., Martin, S. W., and Heatherington, A. C. Clinical dose-response for a broad set of biological products: A model-based meta-analysis. *Statistical Methods in Medical Research*, 27(9):2694–2721, September 2018. ISSN 0962-2802.
